# Lipidome remodeling activities of DPA-EA in palmitic acid-stimulated HepG2 cells and the *in vivo* anti-obesity effect of the DPA-EA and DHA-EA mixture prepared from algae oil

**DOI:** 10.3389/fphar.2023.1146276

**Published:** 2023-03-29

**Authors:** Hua Fang, Yin Cao, Jianyu Zhang, Xiumei Wang, Mengyu Li, Zhuan Hong, Zhen Wu, Meijuan Fang

**Affiliations:** ^1^ Technical Innovation Center for Utilization of Marine Biological Resources, Third Institute of Oceanography, Ministry of Natural Resources, Xiamen, China; ^2^ Fujian Provincial Key Laboratory of Innovative Drug Target Research and State Key Laboratory of Cellular Stress Biology, School of Pharmaceutical Sciences, Xiamen University, Xiamen, China; ^3^ College of Ocean Food and Biological Engineering, Jimei University, Xiamen, China

**Keywords:** DPA-EA, lipidomics, anti-inflammation, anti-obesity, biomarker

## Abstract

**Background:** The nuclear receptor Nur77 has been demonstrated to play a vital role in the inflammatory response and cellular metabolisms, and its ligands exhibit efficacy in the treatment of inflammation-related diseases (e.g., improving mouse acute lung injury (ALI) and obesity. Recently, ω-3 polyunsaturated fatty acid-ethanolamine derivatives (ω-3 PUFA-EAs), including DPA-EA and DHA-EA, have been reported as new Nur77-targeting anti-inflammatory agents. However, the lipid-lowering effect of ω-3 PUFA-EAs is still unknown, and lipid profile changes induced by Nur77-targeting anti-inflammatory agents also remain unclear.

**Objective:** This study aimed to evaluate the lipid-lowering effect and the underlying mechanism of DPA-EA acting as Nur77-targeting anti-inflammatory agents. It also aimed to investigate the *in vitro* and *in vivo* lipid-lowering effects of the DPA-EA and DHA-EA mixture prepared from algae oil.

**Methods:** The *in vitro* lipid-lowing effect of DPA-EA and its mixture with DHA-EA was first evaluated in palmitic acid-stimulated HepG2 Cells. To confirm the lipid-lowering effect and explore the underlying mechanism, we performed untargeted lipidomic analysis using ultra-performance liquid chromatography/triple quadrupole-time-of-flight (TOF) mass spectrometry coupled with multivariate statistical analysis, with another Nur77-targeting anti-inflammatory compound Celastrol (Cel) as a reference. Finally, we examined the anti-obesity effect of the DPA-EA and DHA-EA mixture synthesized from algae oil in a high-fat diet (HFD)-fed mice model.

**Results:** DPA-EA significantly alleviated lipid accumulation with lower toxicity than Celastrol. Nur77-targeting compounds DPA-EA and Celastrol could simultaneously reduce 14 lipids (9 TGs, 2 PCs, 1 PA, 1 SM, and 1 LacCer) and increase 13 lipids (4 DGs, 6 LPEs, 2 PEs, and 1PC) in Pal-stimulated HepG2 cells. However, Cer lipids were more sensitive to DPA-EA, while the over-downregulation of SM lipids might be associated with the off-target toxicity of Celastrol. The mixture of DPA-EA and DHA-EA synthesized from algae oil could significantly decrease TG, TC, and LDL levels and increase HDL levels in HFD-fed mice, exerting an excellent anti-obesity effect.

**Conclusion:** Nur77-targeting anti-inflammatory compound DAP-EA could promote the hydrolysis of PEs and TGs to ameliorate lipid accumulation. The DPA-EA and DHA-EA mixture prepared from algae oil might be a potential therapeutic agent for obesity and other inflammation-related diseases.

## 1 Introduction

Obesity is a complex disease involving abnormal or excessive fat accumulation in subcutaneous and/or organs. Increasing evidence suggests that obesity is a causative factor in developing many chronic inflammation-related diseases (Wang et al., 2018), including non-alcoholic fatty liver disease (NAFLD), cardiovascular disease, type 2 diabetes, colitis, cancer, *etc.* Nowadays, obesity and its complications have become a social health problem affecting the quality of life and even threatening life (Rana et al., 2007). It is demonstrated that excess macronutrients in adipose tissue can stimulate the release of inflammatory mediators such as tumor necrosis factor-alpha (TNF-α) and interleukin 6 (IL-6) (Ellulu et al., 2017). In the high-fat diet (HFD)-induced liver disease, high liver enzyme levels in the blood and liver fat accumulation indicate that prolonged mild inflammation is always associated with obesity (Kim et al., 2012). Overall, obesity is characterized as a state of chronic inflammation in adipose tissue mediated by the secretion of a range of inflammatory cytokines. On the other hand, extracts or compounds from plants with potent anti-inflammatory activity also have significant anti-obesity effects. For instance, fermented lemon peel exhibited an anti-obesity effect on HFD-induced obese mice by modulating the inflammatory response (Pan et al., 2022). Maize extract rich in ferulic acid and anthocyanins prevents HFD-induced obesity in mice by modulating Sirtuin 1 (SIRT1)/AMP-activated protein kinase (AMPK)/IL-6 associated metabolic and inflammatory pathways (Luna-Vital et al., 2020). Hakka stir-fried tea produces anti-obesity and anti-inflammation effects on HFD-induced obese mice model *via* activating the AMPK/acetyl-CoA carboxylase (ACC)/carnitine palmitoyltransferase 1 (CPT1) pathway (Li Q. et al., 2020). All these studies suggest that anti-inflammatory substances have significant lipid-lowering effects and can effectively prevent or improve obesity.

Nur77 is a member of the nuclear receptor subfamily 4A (NR4A) subfamily, which can negatively regulate inflammation and has been identified as a promising therapeutic target for treating inflammation-related diseases (Li et al., 2015). Recently, Nur77 has been demonstrated to play a vital role in cellular metabolisms such as lipid metabolism and glucose metabolism (Deng et al., 2022). For instance, adenovirus-mediated liver-specific Nur77 overexpression accounts for the decrease of triglyceride (TG) and high-density lipoprotein (HDL) levels and the increase of low-density lipoprotein (LDL) levels. Additionally, Nur77 overexpression inhibits the lipogenic transcription factor, sterol regulatory element-binding transcription factor 1c (SREBF-1c) (Pols et al., 2008). Increased expression of Nur77 also reduces macrophage-derived foam cell formation and hepatic lipid deposition, accompanied by downregulation of gene levels of inflammatory molecules, adhesion molecules, and intestinal lipid absorption (Hu et al., 2014). In contrast, Nur77 knockdown increases susceptibility to HFD-induced obesity (Chen et al., 2015). Besides, the liver tissue of Nur77-null mice can accumulate more TGs than wild-type mice (Chao et al., 2009). In particular, Celastrol, a previously reported Nur77 modulator, can not only inhibit acute liver inflammation and chronic inflammation in obese animals (Hu et al., 2017) but also ameliorate NAFLD by decreasing lipid synthesis and improving the anti-oxidative and anti-inflammatory status *via* SIRT1(Zhang et al., 2017). Together, Nur77 serves as a vital therapeutic target in inflammation-related diseases, and its protective effect on improving obesity has been confirmed. Nur77-targeting anti-inflammatory agents may be an effective strategy for improving obesity and its related diseases. However, lipid profile changes induced by anti-inflammatory compounds targeting Nur77 in the lipid-lowering process remain unclear.

Unsaturated fatty acids (UFAs) have broad and critical biological functions (Calder, 2017), (Menegaut et al., 2019). Among them, polyunsaturated fatty acids (PUFA), which can only get from food, are healthy fatty acids that the body needs for brain function and cell growth. Nowadays, much attention has been given to the ω-3 polyunsaturated fatty acids (ω-3 PUFAs), a series of representative natural products with anti-inflammatory and anti-obesity activities. ω-3 PUFAs can affect intracellular signal transduction by elevating the ratio of eicosapentaenoic acid (EPA) and docosahexaenoic acid (DHA) in the cell membrane and inhibiting the transcriptional activity of the inflammation-related transcription factor NF-κB, thereby reducing the pro-inflammatory cytokine TNF-α, IL-6, and interleukin 8 (IL-8) expression (Schumann and Fuhrmann, 2010). Also, ω-3 PUFA can stimulate neutrophils and macrophages to secrete anti-inflammatory cytokines such as interleukin 10 (IL-10), interleukin 4 (IL-4), and interleukin 13 (IL-13) to reduce the inflammatory response (de Bus et al., 2019). Besides,ω-3 PUFAs have been demonstrated to alleviate HFD-induced obesity in mice by reducing adipose tissue cellularity (Ruzickova et al., 2005). Importantly, DHA, an ω-3 PUFA, has been identified as an endogenous lipid reported to bind to Nur77 (Fang et al., 2020). In our recent work, ω-3 polyunsaturated fatty acid-ethanolamine (PUFA-EA) derivatives such as docosahexaenoic acid-ethanolamine (DHA-EA), eicosapentaenoic acid-ethanolamine (EPA-EA), and docosapentaenoic acid-ethanolamine (DPA-EA) were identified as excellent anti-inflammatory agents targeting Nur77 (Fang et al., 2020). Notably, the representative compound DPA-EA (4 k) was demonstrated to bind to Nur77 (TR3) and exerted a Nur77-dependent anti-inflammatory effect through the NF-κB pathway (Fang et al., 2020). To explore the lipid profile changes induced by the anti-inflammatory compounds targeting Nur77, DPA-EA was selected to assess the lipid-lowering effect and underlying mechanism, with Celastrol as a reference compound. Furthermore, given that PUFA-EAs exhibited excellent anti-inflammatory activity and algae oil was a plant-based source of ω-3 PUFA widely used as a dietary supplement, the ω-3 PUFA-EA mixture was synthesized by the reaction of algae oil with ethanolamine under free-solvent condition and evaluated for the potential anti-obesity effect on the HFD-induced obese mice model and the acute toxicity in mice.

## 2 Materials and methods

### 2.1 Reagents and chemicals

DPA-EA was obtained as our previously reported (Fang et al., 2020). Algae oil, composed of 16.23% DHA and 5.22% DPA, was purchased from Xiamen Kingdomway Group Company (Fujian, China). High-performance liquid chromatography (HPLC) grade methanol was purchased from Sigma Aldrich (St. Louis, MO, United States). The HFD was obtained from Nanjing University of Chinese Medical Animal Center, with the composition of 40.56% corn starch, 7.78% wheat meal, 20.23% fish meal, 14.56% oil, 2.33% bone meal, 1.79% yeast powder, 1.39% salt, 0.16% vitamin, 10% egg yolk powder, and 1.2% cholesterol. The mouse IL-6 enzyme-linked immunosorbent assay (ELISA) kit and TNF-α ELISA kit were purchased from DAKEWE (DAKEWE, China). Interleukin-1β (IL-1β) and TNF-α antibodies were purchased from Abcam (Abcam, Cambridge, UK). The kits of TG, TC, LDL cholesterol (LDL-C), and HDL cholesterol (HDL-C) were purchased from Beijing North Kangtai clinical Reagent Co., Ltd., Beijing, China. Saline was prepared as a 0.9% NaCl solution. Griess reagent was purchased from Beyotime (Beyotime, Shanghai, China). MTT (3-(4,5-dimethylthiazol-2-yl)-2,5-diphenyltetrazolium bromide) was purchased from Beyotime (Beyotime, Shanghai, China). Lipopolysaccharide (LPS) was purchased from Sigma (Sigma, St. Louis, MO, United States). Other reagents used in this study were purchased from the domestic market and were all analytically pure and used without further purification.

### 2.2 MTT assay

HepG2 cells were obtained from the American Type Culture Collection (United States) and were cultured in Dulbecco’s Modified Eagle Medium (DMEM) (Hyclone, Logan, Utah, United States) supplemented with 10% FBS, 100 U/mL penicillin, and 100 mg/mL streptomycin at 37°C with 5% CO_2_. MTT assay was performed according to the previously described (Fang et al., 2020). HepG2 cells were seeded at 4.5 × 10^3^ cells per well into 96-well plates and incubated overnight; celastrol or DPA-EA with indicated concentrations was prepared and added to each well, respectively. Following drug treatment for 24 h, 20 μL of MTT solution (5 mg/mL) was added to each well, and the cells were incubated for 4 h at 37°C. Then, the solution containing MTT was removed, and 150 μL of dimethyl sulfoxide was added per well to dissolve the formazan. Finally, the absorbance of the solution was measured at 490 nm (OD490) using a UV spectrophotometer (Model 3555, Thermo). Cell viability was calculated from three independent experiments. The average density of the control group (1‰ DMSO) was set as 100% of viability. Cell viability (%) = compound (OD490)/blank (OD490)×100%. Finally, the IC_50_ value was obtained with Graphpad Prism 8.0.

### 2.3 Lipid accumulation observation

HepG2 cells were inoculated into the 12-well plate at a cell density of 3.0×10^5^/well, and small slides were pre-placed in the bottom of each well in the 12-well plate before inoculating the cells for cell crawling. Cells were adhered overnight, pretreated with 1‰ DMSO or selected compounds at different concentrations for 2 h, and then stimulated with 200 μM palmitic acid (Pal). After 24 h treatment, the supernatant medium was discarded, and the cells were stained with Oil Red O. We detected Pal-induced lipid accumulation in HepG2 cells using the Oil Red O stain kit (ACMEC, AG1262-4).

### 2.4 Lipidomics analysis in HepG2 cell model

#### 2.4.1 Cell sample collection

HepG2 cells were inoculated at ∼10^6^ cells per well in a 10 cm plate and cultured in DMEM for 20 h. The cells in control groups were only treated with 1‰ DMSO. The cells in Pal, Pal+DPA-EA, and Pal+Cel groups were pretreated with 1‰ DMSO, 10 mM DPA-EA, and 2 mM Celastrol for 2h, respectively, then stimulated with 200 μM Pal. After 24 h treatment, the supernatant medium was discarded, and the cells were quickly washed with ice-cold PBS three times, digested with trypsin (HyClone, United States), and performed cell count. After the supernatant was discarded, the pretreat HepG2 cell samples for Control, Pal, Pal + DPA-EA, and Pal + Cel groups (n = 7) were quenched with the following buffer (8.5 g/L NH_4_HCO_3_, 60% MeOH, pH = 7.4). Next, the quenched cell samples were collected by centrifugation (12,000 rpm, 5 min, 4°C) and stored at −80°C before lipid extraction.

#### 2.4.2 Lipid extraction

The extract buffer (methyl tert-butyl ether (MTBE) : methanol = 5:1, 480 μL) containing internal standard was added to the samples, then frozen and thawed with liquid nitrogen for 3 times. Next, the cell samples were sonicated for 10 min in an ice-water bath, incubated at −40°C for 1 h, and centrifuged at 3000 rpm for 15 min at 4°C. Approximately 350 μL of supernatant was transferred to a fresh tube and dried in a vacuum concentrator at 37°C. Subsequently, the dried samples were reconstituted in 100 μL of 50% methanol in dichloromethane by sonication for 10 min in the ice-water bath. The constitution was then centrifuged at 13,000 rpm for 15 min at 4°C, and 80 μL of supernatant was transferred to a fresh glass vial for LC/MS analysis. The quality control (QC) sample was prepared by mixing an equal aliquot of the supernatants from all of the samples and was used to evaluate the stability and reproducibility of the analytes detected in the analytical run. The lipid extraction experiment was done by Biotree Biotech Co., Ltd (Shanghai, China).

#### 2.4.3 Lipid detection by UPLC-HRMS/MS

The UPLC separation was conducted using a 1290 Infinity series UPLC System (Agilent Technologies), equipped with a Kinetex C18 column (2.1 * 100 mm, 1.7 μm, Phenomen). The mobile phase was a combination of solvent A (40% water, 60% acetonitrile supplemented with 10 mmol/L ammonium formate) and solvent B (10% acetonitrile, 90% isopropanol added with 5% 10 mmol/L ammonium formate). The analysis was carried out with a linear gradient as follows: 0–12.0 min, 40%–100% B; 12.0–13.5 min, 100% B; 13.5–13.7 min, 100%–40% B; 13.7–18.0 min, 40% B. The column temperature was 45°C. The auto-sampler temperature was 4°C, and the injection volume was 0.5 μL in positive mode or 2 μL in negative mode. The Triple TOF mass spectrometer (Triple TOF 6600, ABsciex) was used for its ability to acquire MS/MS spectra on an information-dependent basis (IDA) during an LC/MS experiment. In each cycle, 12 precursor ions with the strongest intensities above 100 were chosen for MS/MS at collision energy of 45 eV. ESI source conditions were set as follows: Gas 1: 60 psi, Gas 2: 60 psi, Curtain Gas: 30 psi, Source Temperature as 600°C, Declustering potential as 100 V, Ion Spray Voltage Floating (ISVF) as 5,000 V or −4,500 V in positive or negative modes, respectively. Full scan was conducted along with the MS/MS and the *m/z* scan range was set as 200–2,000. The UPLC-HRMS/MS analysis was assisted by Biotree Biotech Co., Ltd (Shanghai, China).

#### 2.4.4 Data preprocessing and annotation

The raw data files (.wiff format) were converted to files in mzXML format with ProteoWizard. XCMS was applied for retention time correction, peak identification, peak extraction, peak integration, and peak alignment with minfrac set to 0.5 and cutoff set to 0.6. Lipid identification was performed using XCMS-based software, a self-written R package, and a self-built lipid secondary database. Furthermore, all processed peak areas of lipid substances (see Supplementary Data S1) were imported into SIMCA-P14.1 software (Umetrics) for multivariate statistical analysis, including principal component analyses (PCA), orthogonal projections to latent structures discriminate analysis (OPLS-DA), and permutation tests. Lipid substances with a VIP value > 1.0 in the OPLS-DA model and a *p*-value <0.05 in the Student’s t-test were considered important differential biomarkers for discriminating each group.

### 2.5 The preparation of the PUFA-EA mixture from algae oil

The DPA-EA and DHA-EA mixture was prepared as described in Supplementary Scheme S1. Algae oil (100.0 g) and ethanolamine (15.0 g) were added to a round bottom flask (250 mL) without any solvent. The reaction mixture was stirred for 24 h at 80°C, which was purified by silica gel column chromatography (petroleum ether/ethyl acetate 1:1) to give PUFA-EA mixture as pale yellow oil (14.1 g). The reverse phase HPLC (C18, methanol/H_2_O = 90:10) analysis indicated that the mixture contained 67.54% of DHA-EA (tR = 6.58 min) and 27.96% of DPA-EA (tR = 7.65 min), respectively (Supplementary Scheme S1 and Supplementary Figure S1).

### 2.6 Anti-inflammatory evaluation in RAW264.7 cells

Mouse RAW 264.7 macrophages were obtained from the American Type Culture Collection (United States). Cells were treated with DPA-EA, DHA-EA and EPA-EA with the concentration of 10 μM and PUFA-EA with gradient dose from 20 to 2.5 μM. The experiments, including NO production assay, Real-Time Quantitative PCR, and Western blot Analysis, were performed as described previously (Fang et al., 2020).

### 2.7 Determination of anti-obesity activity *in vivo*

In total, 40 ICR (male/female = 1:1, 18–22 g) mice were obtained from Nanjing University of Chinese Medical Animal Center. The animal room was maintained at 22°C ± 2°C and humidity-controlled 55% ± 5% with a 12-h light and 12-h dark cycle. After a 3-day acclimatization period, feeding with a standard rat diet, forty mice were divided randomly into four groups (male/female = 1:1): The normal diet (ND) group was the normal control in which mice were fed with a standard rat diet. The HFD group was the model group in which mice were fed with an HFD. The HFD + ORL group was the positive control group in which mice were fed an HFD and intragastric administration with Orlistat (ORL) at 0.468 g/kg daily. The HFD + PUFA-EA group was a treatment group in which mice were fed with an HFD and intragastric administration with algae oil-derived PUFA-EA mixture at 0.167 g/kg daily. Drugs were intragastrically administered as a suspension in 0.5% carboxymethyl cellulose for 36 consecutive days. The ND and HFD groups were administered only the 0.5% carboxymethyl cellulose vehicle. The food and water were available *ad libitum* during the 6-week administration. This experiment was performed in compliance with the Chinese legislation on the use and care of laboratory animals and was approved by the Experimental Animals Ethics Committee of Nanjing University of Chinese Medicine (No. 201906A012).

### 2.8 Determination of serum biomarkers

The serum levels of TG, TC, LDL-C, and HDL-C were measured enzymatically with commercial assay kits. TC content was determined with the total cholestenone content assay kit (Solarbio, BC 1985), and TG was measured with a triglyceride content detection kit (Solarbio, BC0625). The assay kits of LDL-C and HDL-C were BC5335 and BC5325 from Solarbio, respectively. The results were expressed as mmol/L. All analytical procedures follow the essay kit’s instructions and are replicated at least three times.

### 2.9 Morphological analysis of hepatic tissue

Liver samples removed from mice were fixed overnight with 4% paraformaldehyde. Fixed tissues were embedded in paraffin, sliced into 3 μm pieces, and stained with hematoxylin and eosin (H&E). The stained areas were viewed using an optical microscope (Olympus CX31, Tokyo, Japan) with a magnifying power of ×200.

### 2.10 Acute toxicity study in mice

Acute toxicity studies were carried out according to OECD Test Guidelines. The C57BL/6 mice used (8–10 weeks old and 18–20 g) were provided by the Animal Experiment Center of Xiamen University, Fujian, China. After the mice were adaptively reared in the animal room for 5 days, the mice (20 males and 20 females) were randomly divided into four groups (control mice: male (10), female (10). Mice treated by PUFA-EA: male (10) and female (10)). The 0.6 mL (540 mg) PUFA-EA was intragastrically administrated as suspension in 0.5% carboxymethyl cellulose twice a day for 14 days consecutively in the treatment group, and the control group was intragastrically administrated with 0.6 mL saline. The maximum tolerated dose is calculated as 540 × 2/0.02 = 54,000 mg/kg. Mice had free access to distilled water and commercial standard diets. Feeding behaviors such as food intake and drinking water were measured daily. General appearances (spirit, skin, fur, eyes, nose, respiration, urine, feces, and locomotor) were observed daily. This experiment was performed in compliance with Chinese legislation on the use and care of laboratory animals. This animal experiment was approved by the animal care and use committee of Xiamen University (XmuLAC 20200029).

### 2.11 Statistical analysis

All assays were performed in triplicate. The result’s data were expressed as mean ± SD (standard deviation). Other data were analyzed by the SPSS statistical software, version 18.0 (SPSS Inc., United States). Student’s t-test was used as specified in the figure legends. P < 0.05 was considered statistically significant (*), *p* < 0.01 as highly significant (**), *p* < 0.001 as extremely significant (***), and ns as not significant.

## 3 Results

### 3.1 Evaluation of the lipid-lowering activity of Nur77 modulators in Pal-stimulated HepG2 cells

**FIGURE 1:**
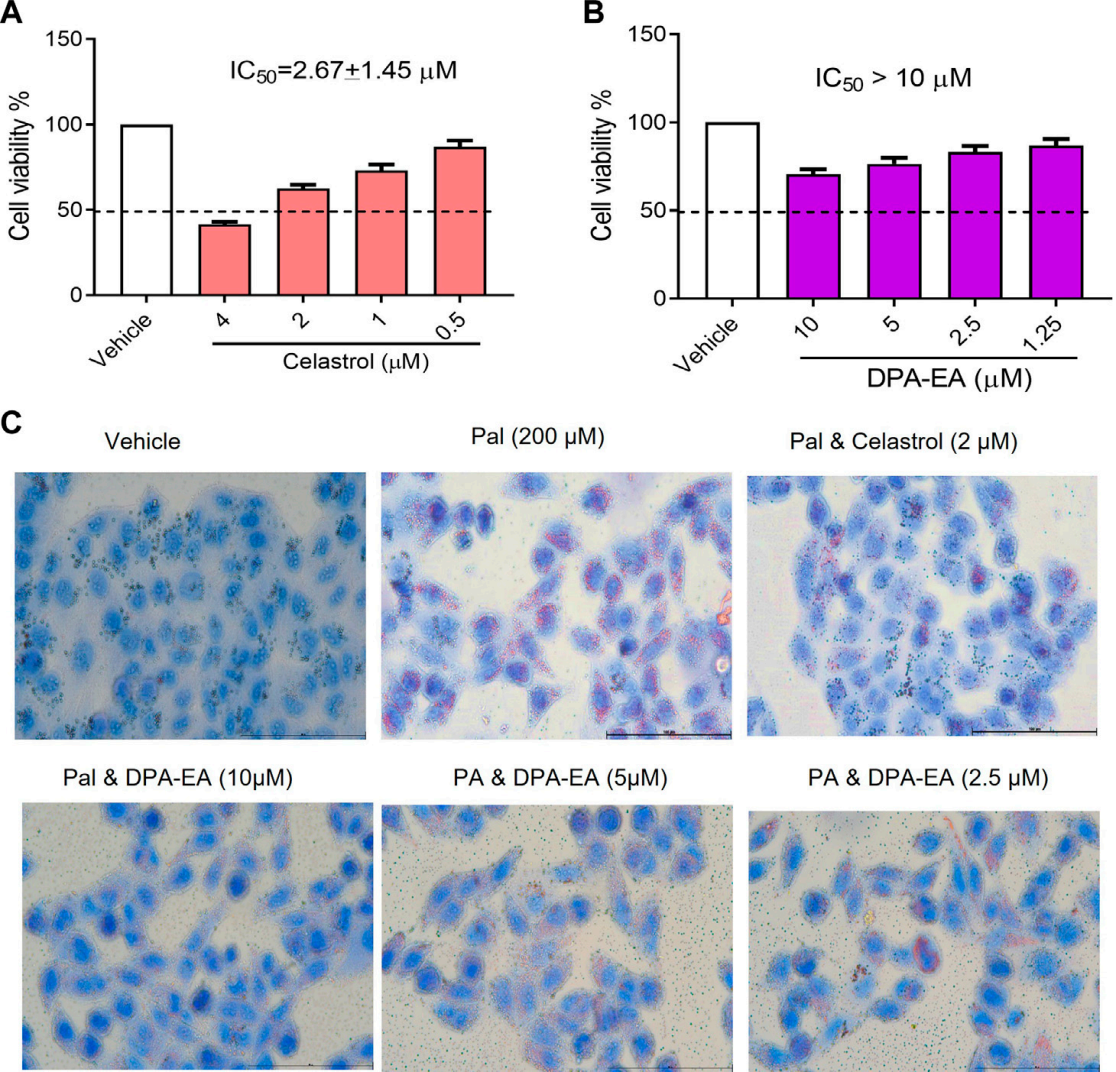
Effects of Celastrol and DPA-EA on cell viability and lipid droplets in HepG2 cells. **(A)** Effects of Celastrol at different concentrations (0.5, 1, 2, and 4 μM) on the cell growth of HepG2. **(B)** Effects of DPA-EA at different concentrations (1.25, 2.5, 5, and 10 μM) on the cell growth of HepG2 cells. **(C)** Effects of DPA-EA and Celastrol at indicating concentrations on formation of lipid droplets in Pal-induced (200 μM) HepG2 cells with Oil red staining assay. Images were captured with a microscope (200×).

NAFLD is a common inflammation-related disease. Its occurrence is mainly due to the body’s metabolic disorders, along with the accumulation of liver fat in the body. Nur77 plays diverse and vital roles in regulating lipid metabolism and emerges as a hot therapeutic target for metabolic diseases (Chao et al., 2009). Celastrol has been reported to be a modulator of Nur77 and exerts anti-inflammation, lipidome remodeling, and weight-losing function (Zhang et al., 2017; Wang et al., 2021). Interestingly, we have demonstrated that PUFA-EAs such as DPA-EA and DHA-EA exert excellent anti-inflammation activities in a Nur77-dependent manner (Fang et al., 2020). Herein, we constructed an adipose hepatocyte model by Pal-induced HepG2 lipid accumulation to detect the lipid-lowering activity of DPA-EA, with Celastrol as a reference compound. The cytotoxicity of DPA-EA and Celastrol against HepG2 cells was first tested using the MTT assay. DPA-EA at 10 μM reduced cell growth to 71.7% (Figure 1A), exhibiting low cell growth inhibition toward HepG2 (IC_50_ > 10 μM). Celastrol displayed noticeable cytotoxic activity with an IC_50_ value of 2.67 μM (Figure 1B). Then, the effect of DPA-EA and Celastrol on lipid accumulation was observed under a microscope using an oil-red O staining experiment. The results are shown in Figure 1C and Supplementary Figure S2. Pal significantly caused lipid accumulation in HepG2 because an apparent increase of red oil droplets was observed under the microscope. Celastrol treatment decreased red oil droplets, confirming the lipid-lowering effects of Celastrol (Liu et al., 2015). Also, DPA-EA attenuated Pal-induced lipid accumulation in HepG2, with a dose-dependent reduction in red lipid droplets. In contrast, DPA-EA at 10 and 5 µM showed better lipid-lowering effects than celastrol at 4 μM, with considerably lower cytotoxicity (Figure 1 and Supplementary Figure S2). These results suggested that DPA-EA might be an effective lipid-lowering compound with lower cytotoxicity than Celastrol.

### 3.2 Perturbation of DPA-EA and celastrol on the lipid profile of pal-induced HepG2 cells

#### 3.2.1 Raw mass spectrometry data preprocessing

The experimental groups were set as followings: untreated hepatocyte group (Ctrl group); Pal-stimulated HepG2 group (Pal group); Pal-stimulated HepG2 cells with DPA-EA intervention (Pal + DPA-EA group); and Pal-stimulated HepG2 cells with Celastrol administration (Pal + Cel group). The lipid metabolites in each group were examined using UHPLC-QTOF-MS technology. After baseline filtering, peak recognition and integration, retention time correction, peak alignment, and normalization to an internal standard (IS), we detected a total of 21 major lipids, including TG, diacylglycerol (DG), monoglyceride (MG), cholesteryl ester (CE), phosphatidylcholine (PC), phosphatidylethanolamine (PE), phosphatidic acid (PA), phosphatidylinositol (PI), phosphatidylserine (PS), phosphatidylglycerol (PG), lysophosphatidylcholine (LPC), Lysophosphatidylethanolamines (LPE), lysophosphatidylinositol (LPI), lysophosphatidylserine (LysoPS), lysophosphatidic acid (LPA), lysophosphatidylglycerol (LPG), ceramide (Cer), glucosylceramide (GlcCer), lactosylceramide (LacCer), sphingomyelin (SM), and sphingosine (Sph) in HepG2 cells. Besides, 990 and 711 lipids were detected in the positive and negative ion patterns, respectively. The QC procedures showed good reproducibility of the collected data in the positive and negative ion modes (Supplementary Figures S3A, B). Besides, PCA models demonstrated the excellent aggregation of QC samples (Supplementary Figures S3C, D), suggesting the stable instrument state and high reliability of the experimental data.

#### 3.2.2 The differential lipid metabolite profile of HepG2 cells under Pal stimulation

**FIGURE 2:**
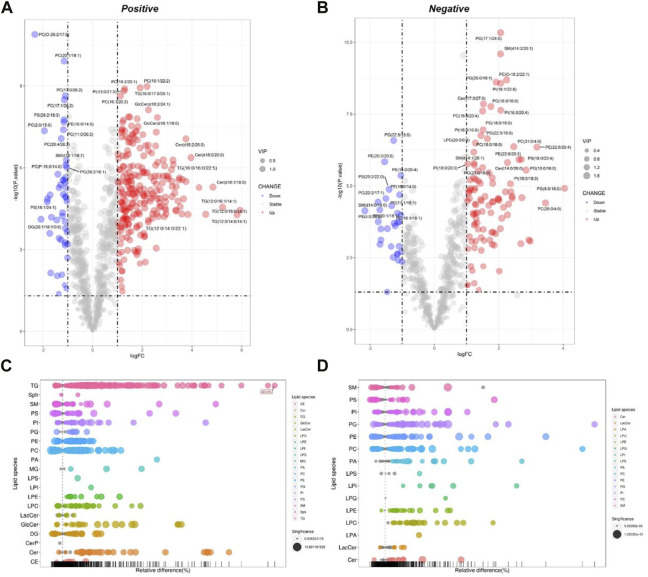
Analysis of significantly differential lipid substances in the Pal group *versus* the Ctrl group. Volcano plots **(A,B)** and lipid plots **(C,D)** in both positive and negative ion modes, respectively. In the volcano plot, lipid species were screened out based on the criteria of *p* < 0.05, VIP≥1, and fold change <0.5 or >2.

The processed MS data were imported into SIMCA 14.0 software (Umetrics) for multivariate pattern recognition analysis. The PCA analysis showed that all samples were within the 95% confidence interval in both positive and negative ion modes, and significant existed differences between the Ctrl and the Pal groups (Supplementary Table S1 and Supplementary Figures S4A, B). To maximize the distinction between the Ctrl and Pal groups, OPLS-DA analysis was also executed for the collected data in positive and negative ion modes, respectively. It can be observed that there was a significant lipid metabolism change between the Ctrl and Pal groups (Supplementary Figures S5A, B), and the corresponding model parameters were in the effective range (Table 1). These results indicated that Pal stimulation caused significant alterations in the lipid profile of HepG2 hepatocytes. According to *p* < 0.05 and VIP >1, we first picked out 571 and 363 differential-expressed lipids (DELs) in the positive and negative ion modes, respectively (Supplementary Table S2). Totally, Pal stimulation in HepG2 cells led to 865 DELs. Most of them (563) were upregulated. Using the fold change (FC) value of >2 as a cutoff, we found that Pal significantly upregulated the expression of 310 lipids, including TG (103), PC (34), Cer (32), PE (20), *etc* (Supplementary Table S4, Figures 2A, B). Notably, TG (12:0/16:0/14:1), TG (12:0/14:0/14:1), TG (12:0/16:1/14:1), Cer(d18:1)/18:0) and SM (d14:1/20:0) were significantly elevated in Pal-stimulated HepG2 cells (Figures 2A, B), with the fold change indices (Pal/Ctrl) of 61.13, 56.33, 37.54, 28.75, and 19.46, respectively. These results were consistent with lipid droplet accumulation in Pal-stimulated HepG2 cells. Additionally, we found 302 Pal-downregulated DELs. Using the FC value of <1/2 as a cutoff, we observed that Pal could significantly downregulate the expression of 71 lipids involved in 21 lipid species of six classes, in which glycerophospholipids (GPs) were identified as the most species (58), such as PC (36) and PS (13) (Supplementary Table S3 and Figures 2C, D). Especially, PC (O-20:2/17:0), PC (2:0/15:0), PS (4:0/26:0), and PS (18:1/24:1) expressions were significantly downregulated (Figures 2A, B), with the FC values of 0.20, 0.26, 0.22 and 0.28, respectively. Interestingly, several DGs were also observed with a robust reduction, especially DG (26:1/18:1/0:0) and DG (24:1/18:1/0:0) with corresponding FC indices of 0.24 and 0.28 (Figures 2A, B).

**TABLE 1:** Corresponding values of R^2^X, R^2^Y, and Q^2^ of OPLS-DA models.

*No.*	Description	Ion mode	N	R^2^X (cum)	R^2^Y (cum)	Q^2^ (cum)	R^2^Y - Q^2^
1	Ctrl, Pal, Pal + DPA-EA, and Pal + Cel	(+)	28	0.774	0.988	0.972	0.016
		(−)	28	0.783	0.980	0.961	0.019
2	Pal vs. Ctrl	(+)	14	0.683	0.994	0.980	0.014
		(−)	14	0.741	0.980	0.968	0.012
3	Pal + Cel vs. Pal	(+)	14	0.672	0.992	0.969	0.023
		(−)	14	0.689	0.987	0.963	0.024
4	Pal + DPA-EA vs. Pal	(+)	14	0.630	0.996	0.977	0.019
		(−)	14	0.659	0.989	0.963	0.026

Ctrl, Control group; Pal: Pal-induced HepG2 group; Pal + Cel: Pal-stimulated HepG2 cells with Celastrol administration; Pal + DPA-EA: Pal-stimulated HepG2 cells with DPA-EA, intervention. *Vs, versus*.

#### 3.2.3 Lipid profile’s perturbation by Nur77 modulators in Pal-induced fatty liver HepG2 cells

##### 3.2.3.1 Celastrol-induced lipidomics changes in Pal-stimulated HepG2 cells

**FIGURE 3:**
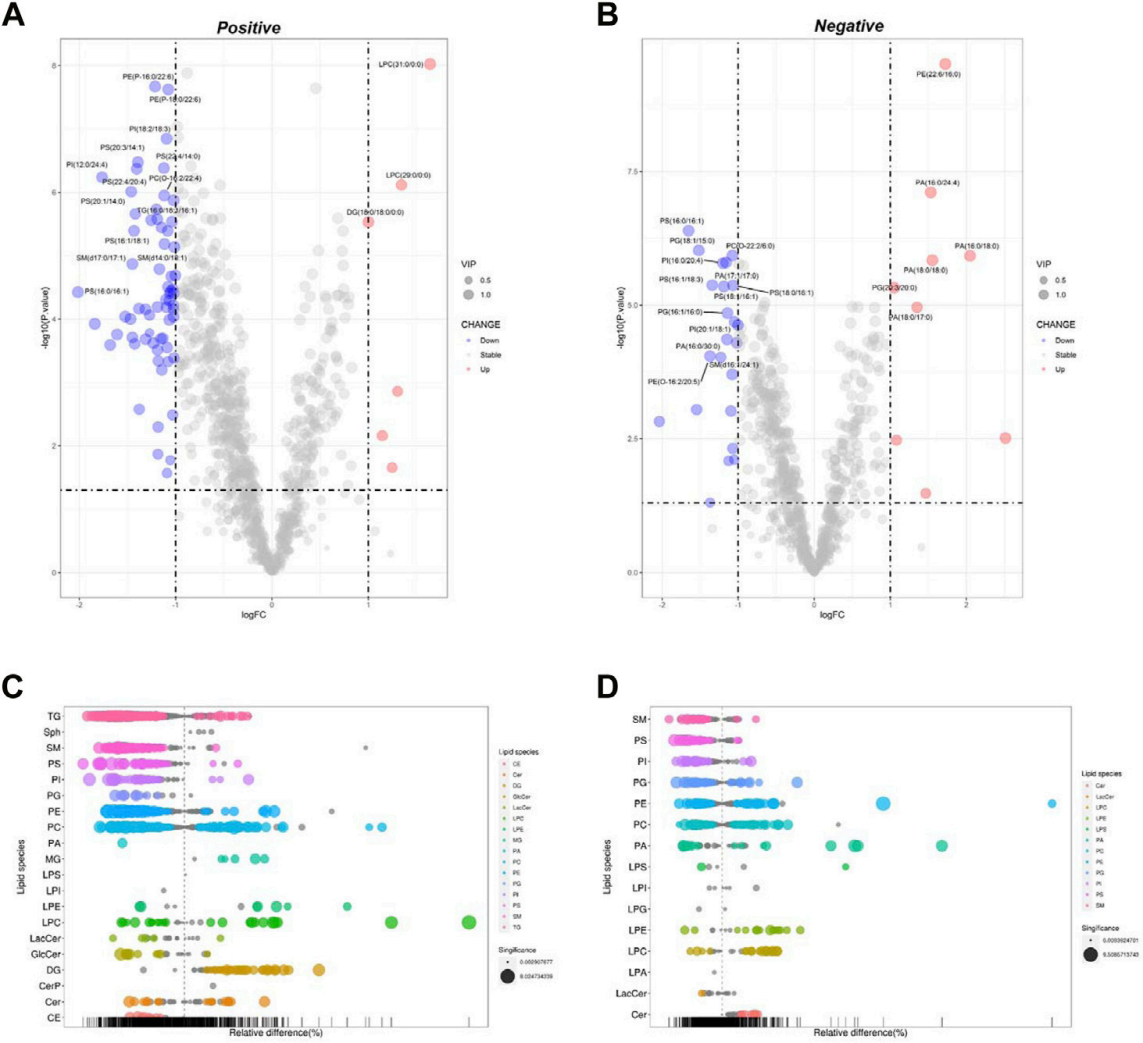
Analysis of significantly differential lipid substances in the Pal + Cel group *versus* the Pal group. Volcano plots **(A,B)** and lipid plots **(C,D)** in both positive and negative ion modes, respectively. In the volcano plot, lipid species were screened out based on the criteria of *p* < 0.05, VIP≥1, and fold change <0.5 or >2.

Celastrol is a well-known Nur77 modulator with an excellent weight-loss effect in HFD-fed mice (Hu et al., 2017). Also, our oil-red O staining experiment showed that Celastrol decreased Pal-induced lipid accumulation in HepG2 cells. Therefore, we treated Pal-stimulated HepG2 cells with Celastrol for 24 h and then performed lipidomics analysis to explore whether Celastrol could reprogram the hepatic lipidome. PCA score plots indicated significant differences in lipid profiles between the Pal + Cel and Pal groups in the positive and negative ion modes (Supplementary Table S2, Supplementary Figures S4C, D), suggesting that Celastrol intervention could significantly alter the lipid profile of Pal-stimulated HepG2 cells. Next, we performed an OPLS-DA analysis to gain further insight into global lipid alterations (Supplementary Figures S5C, D). Two separate multivariate OPLS-DA models of Pal + Cel and Pal groups were generated for the positive and negative ion patterns. Their relevant validation parameters all satisfied R^2^Y > 0.9, Q^2^ > 0.9, and R^2^Y - Q^2^ < 0.2, indicating a faithful representation of the data and a good predictive ability of the models (Table 1). The good separations of the Pal and Pal + Cel groups in both models implied that Celastrol could alleviate lipid metabolism disorders to a certain extent. According to *p* < 0.05 and VIP >1, we picked out 469 and 326 DELs between the Pal + Cel and Pal groups in the positive and negative ion modes, respectively (Supplementary Table S2). In total, Celastrol administration in Pal-stimulated HepG2 cells led to 722 DELs. Notably, Celastrol could significantly decrease 78 lipids (FC < 1/2) in Pal-stimulated HepG2 cells, mainly including TG (23), PC (11), PI (11), SM (11), PS (9), et al. (Supplementary Table S3, Figures 3A–D). Typically, Celastrol downregulated PS(16:0/16:1), TG (16:1/16:1/16:1), PI(12:0/24:4), TG (12:0/14:0/14:1), and TG (14:0/16:1/16:1) with the FC indices of 0.24, 0.27, 0.29, 0.31 and 0.32, respectively. Meanwhile, the expression levels of 15 lipids significantly increased (FC > 2) in the Pal + Cel group compared to the Pal group (Supplementary Table S3, Figures 3A, B). Significantly, Celastrol administration elevated the contents of LPC(31:0/0:0), LPC(29:0/0:0), PE (5:0/18:0), and PA (16:0/18:0) with the FC indices of 3.11, 2.54, 5.71, and 4.14, respectively.

##### 3.2.3.2 DPA-EA-induced lipidomics changes in Pal-stimulated HepG2 cells

**FIGURE 4:**
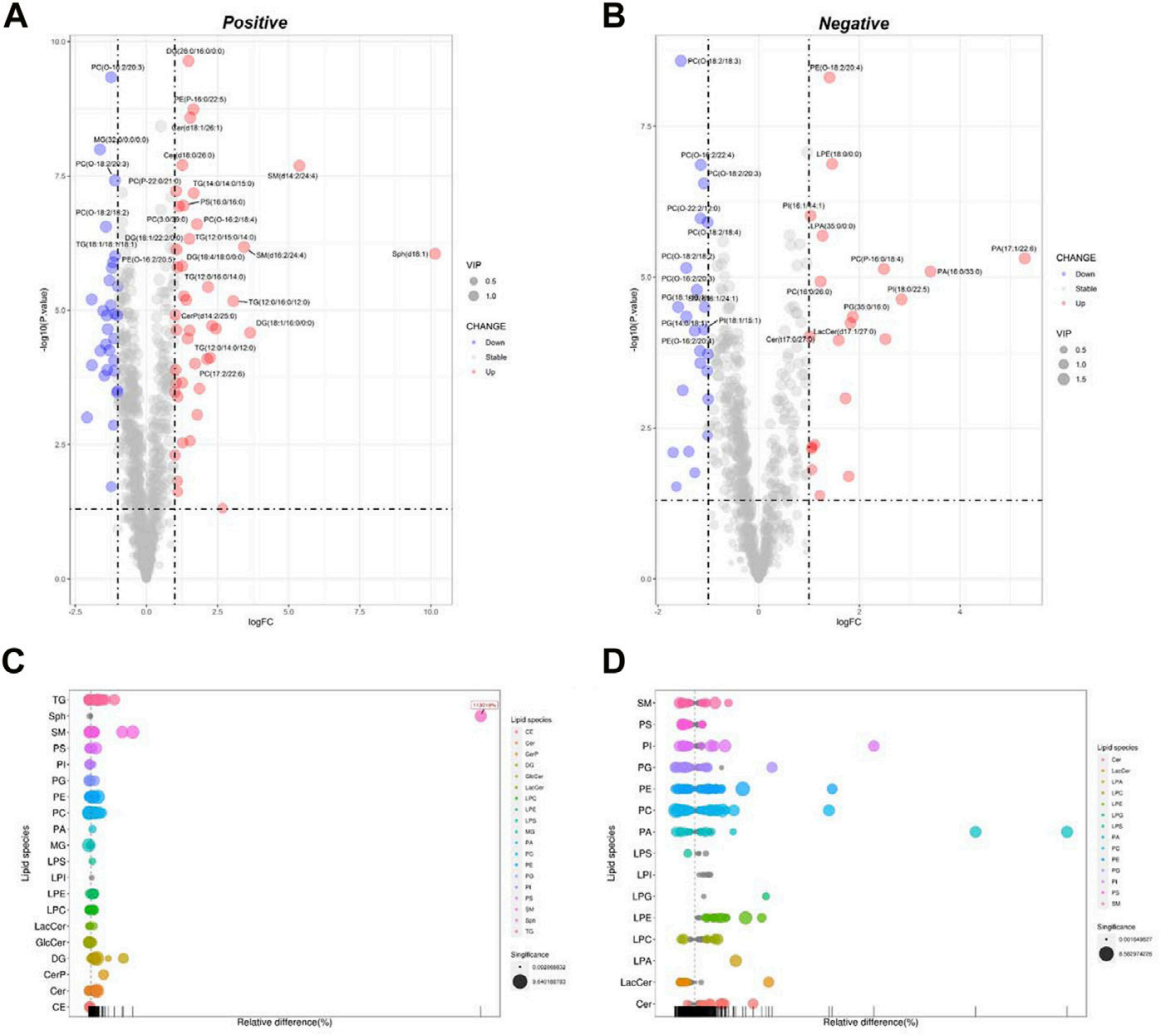
Analysis of significantly differential lipid substances in the Pal + DPA-EA group *versus* the Pal group. Volcano plots **(A,B)** and lipid plots **(C,D)** in both positive and negative ion modes, respectively. In the volcano plot, lipid species were screened out based on the criteria of *p* < 0.05, VIP≥1, and fold change <0.5 or >2.

DPA-EA is a potent anti-inflammatory compound targeting Nur77 that we previously reported (Fang et al., 2020). Furthermore, we also demonstrated that DPA-EA could alleviate Pal-induced lipid accumulation in HepG2 cells. Therefore, we treated Pal-stimulated HepG2 cells with DPA-EA for 24 h and analyzed the interference of DPA-EA on lipidomics profiling of Pal-stimulated HepG2 cells to understand the lipid-lowering mechanism of DPA-EA comprehensively. First, two separate sample clusters, which corresponded to the Pal and Pal + DPA-EA groups, were observed in PCA score plots of positive and negative modes (Supplementary Table S1, Supplementary Figures S4E, F). It signified that DPA-EA might have a significant improvement effect on Pal-induced HepG2 cells at the lipid level. Then, the supervised OPLS-DA models were further constructed to capture the distinctive metabolic phenotypes and to maximize the discrimination between the Pal and Pal + DPA-EA groups. As shown in Table 1 and Supplementary Figures S5E, F, the Pal + DPA-EA group was discriminated from the Pal group with R^2^Y > 0.9, Q^2^ > 0.9, and R^2^Y-Q^2^ < 0.2 in both OPLS-DA models of positive and negative modes. It suggested that DPA-EA could reprogram the lipid profile in Pal-stimulated HepG2 cells. Based on VIP >1.0 and *p* < 0.05, 705 DELs were identified between the Pal + DPA-EA and Pal groups (Supplementary Table S2). Compared with the Pal group, 45 lipids were significantly decreased (FC < 1/2), and 61 lipids were significantly elevated (FC > 2) in the Pal + DPA-EA group (Supplementary Table S4, Figures 4A–D). For example, DPA-EA could significantly reduce the levels of PC(O-18:2/18:3), GlcCer(d18:1/18:0), TG (16:1/16:1/16:1), and TG (16:1/16:1/18:1), with the FC indices of 0.23, 0.26, 0.26, and 0.32, respectively. Meanwhile, DPA-EA robustly increased some lipids such as Sph (d18:1), SM(d14:2/24:4), SM(d16:2/24:4), and PA (17:1/22:6)*,* with the FC indices of 1131.19, 41.59, 10.79, and 39.03, respectively.

#### 3.2.4 Comparison of lipidomics prfile changes caused by DPA-EA and celastrol on Pal-induced fatty liver HepG2 cells

**FIGURE 5:**
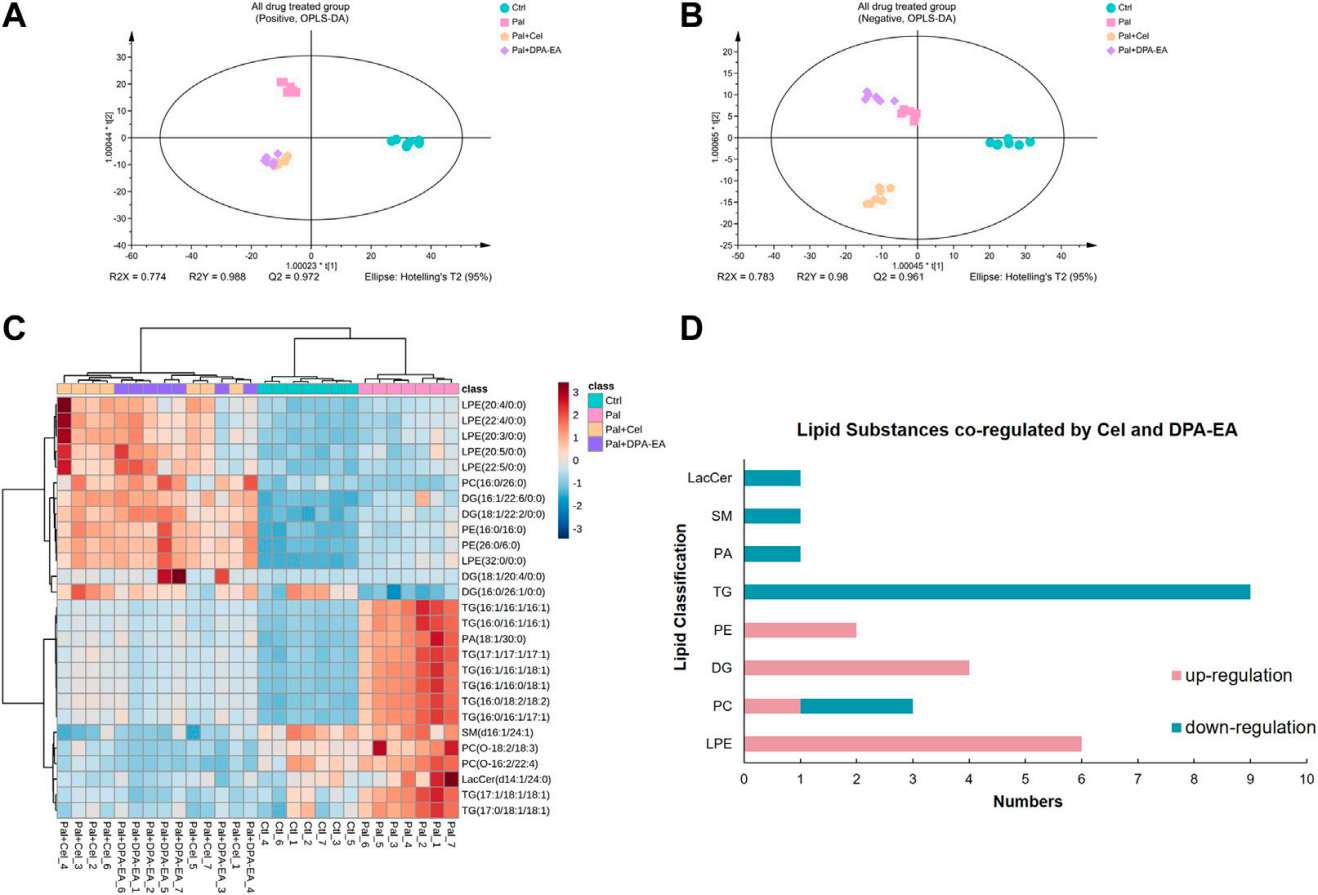
Analysis of significantly differential lipids in Ctrl, Pal, Pal + Cel, and Pal + DPA-EA groups. OPLS-DA score plots **(A,B)** in positive and negative ion modes, respectively. **(C)** Heatmap of common DELs in the Pal + Cel vs. Pal and Pal + DPA-EA vs. Pal models, selected with the fold change cutoff of FC > 1.5 or FC < 0.5 as upregulated and downregulated, respectively. Red and blue indicate increased and decreased lipids, respectively. **(D)** Classifications of the regulated lipid species.

DPA-EA and Celastrol are two Nur77-targeting anti-inflammatory compounds with prominent lipid-lowering activities (Fang et al., 2020). However, DPA-EA showed lower toxicity, while Celastrol had apparent off-target toxicity (Wang et al., 2011). Celastrol and DPA-EA might activate other targets besides Nur77. Therefore, we performed PCA and OPLS-DA analysis among four groups. The Pal + DPA-EA and Pal + Cel groups partially overlapped in PCA analysis (Supplementary Figures S4G, H), hinting that lipid profiles reprogrammed by DPA-EA and Celastrol were part in common. However, the sample clusters of the Pal + DPA-EA group and Pal + Cel group were significantly separated in both positive and negative OPLS-DA score plots (Figures 5A, B). The same DELs caused by DPA-EA and Celastrol interventions may be associated with their Nur77-targeting lipid-lowering effect. In contrast, the different DELs may be caused due to their targeting of other proteins.

##### 3.2.4.1 The same DELs caused by DPA-EA and Celastrol interventions

Between the two multivariate OPLS-DA models (Pal + Cel group vs. Pal group and Pal + DPA-EA group vs. Pal group), there were a total of 443 same DELs (*p* < 0.05 and VIP >1) (Supplementary Table S4). Among them, 272 lipids were simultaneously decreased by DPA-EA and Celastrol administrations (Supplementary Table S4). Notably, the levels of 14 lipids (9 TGs, 2 PCs, 1 PA, 1 SM, and 1 LacCer) were significantly reduced (compared with the PA group, FC < 0.5) both in the Pal + DPA-EA and Pal + Cel groups (Figures 5C, D). These 14 lipids were significantly increased in the Pal group (compared with the Ctrl group); these increases were markedly diminished by supplementation with DPA-EA and Celastrol. Most of these 14 lipids were TGs such as TG (16:1/16:1/16:1) and TG (16:0/16:1/16:1), implying that DPA-EA and Celastrol had the effect of improving lipid metabolism. Besides, DPA-EA and Celastrol could simultaneously upregulate 104 lipids in Pal-stimulated HepG2 cells, mainly concentrated in Cer, DG, PE and PC classes (Supplementary Table S4). Among them, 13 lipids (6 LPEs, 4 DGs, 2 PEs, and 1 PC) were remarkably increased (compared with the Pal group, FC > 1.6) in the Pal + DPA-EA and Pal + Cel groups (Figures 5C, D). Most of these 13 upregulated lipids were LPEs (*e.g.,* LPE (22:5/0:0) and LPE (32:0/0:0)) and DGs (*e.g.,* DG (18:1/20:4/0:0) and DG (16:1/26:1/0:0)). LPEs and DGs are the downstream hydrolysis products of PEs and TGs, respectively. Thus, our results indicated that the common lipid-lowering mechanism of DPA-EA and Celastrol might be by promoting the hydrolysis of esters, thereby reducing the level of esters and alleviating lipid accumulation.

##### 3.2.4.2 The specific DELs caused by Celastrol intervention

**FIGURE 6:**
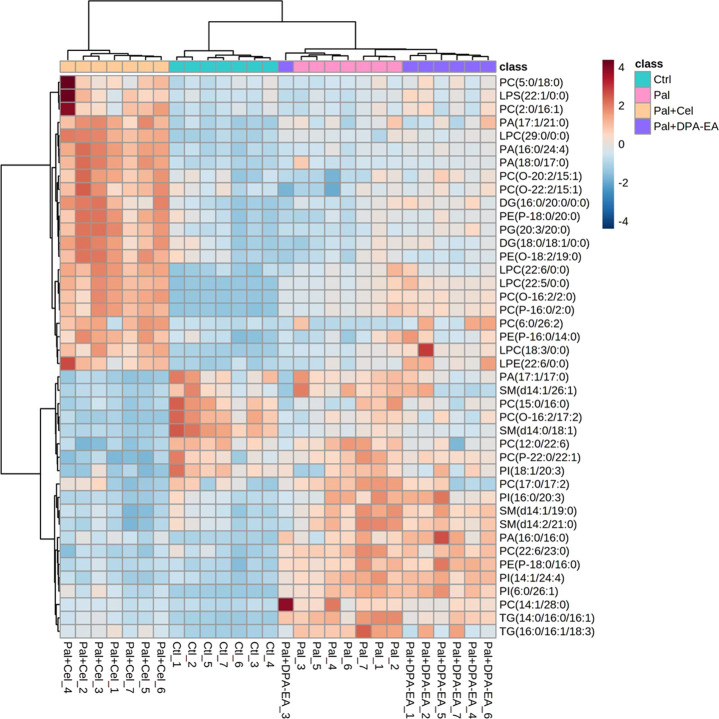
Heatmap of DELs only in the Pal + Cel vs. Pal model, not in the Pal + DPA-EA vs. Pal model, selected with the fold change cutoff of FC > 1.6 or FC < 0.5 as upregulated and downregulated, respectively. Red and blue indicate increased and decreased lipids, respectively.

A total of 722 DELs (VIP >1.0 and *p* < 0.05) were identified for the Pal + Cel group vs. Pal group model, in which 279 DELs could be attained only in the Pal + Cel group vs. Pal model but not in the Pal + DPA-EA vs. Pal model. Interestingly, 200 lipids were downregulated, and 79 lipids were on the contrary (Supplementary Table S5). Notably, 20 lipids were significantly decreased (compared with the Pal group, FC < 0.5), and 22 lipids were significantly increased (compared with the Pal group, FC > 1.6) in the Pal + Cel group (Figure 6). Most of these up- or downregulated DELs belonged to GP class such as PC, LPC, and PE (Figure 6). The first six downregulated lipids with the most significant differences (Pal + Cel group vs. Pal group model) were PI(16:0/20:3), PC(22:6/23:0), TG (14:0/16:0/16:1), PI(14:1/24:4), PC(15:0/16:0) and PC(14:1/28:0), and their average contents in the Pal + Cel group were similar to those in the Ctrl group. At the same time, PA (16:0/24:4), PA (18:0/17:0), LPC (22:5/0:0), PC(6:0/26:2), PC(2:0/16:1), and PG (20:3/20:0) levels were still significantly increased (compared with the Pal group) in the Pal + Cel group and well above those in the Ctrl group.

##### 3.2.4.3 The specific DELs caused by DPA-EA intervention

**FIGURE 7:**
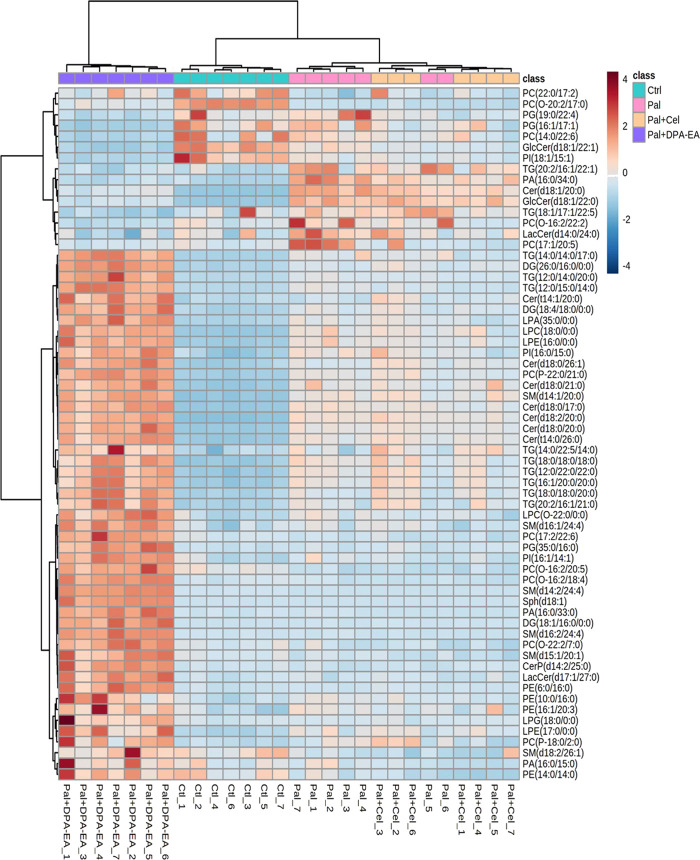
Heatmap of DELs only in the Pal + DPA-EA vs. Pal model, not in the Pal + Cel vs. Pal model, selected with the fold change cutoff of FC > 1.6 or FC < 0.5 as upregulated and downregulated, respectively. Red and blue indicate increased and decreased lipids in each group, respectively.

A total of 705 DELs (VIP >1.0 and *p* < 0.05) were identified for the Pal + DPA-EA vs*.* Pal model. Besides the 443 co-regulated DELs, DPA-EA also individually upregulated 108 lipids (51 lipids with FC value > 1.6) and downregulated 154 lipids (13 lipids with FC value <0.5) (Supplementary Table S6). The 64 lipids with FC > 1.6 or FC < 0.5 were involved in 18 lipid types, including Cer, SM, TG, PE, PC, PG, PI, LPC *et al.* (Figure 7). The types of lipids specificly modulated by DPA-EA were much more than those only modulated by Celastrol (Figures 6, 7). The first six downregulated lipids (the Pal + DPA-EA group vs. Pal group model) were PC(17:1/20:5), PA (16:0/34:0), PC(O-16:2/22:2), PG (19:0/22:4), GlcCer(d18:1/22:1) and GlcCer(d18:1/22:0). Among them, the average contents of GlcCer(d18:1/22:1), PA (16:0/34:0), and PC(O-16:2/22:2) in the DPA-EA group were similar to those in the Ctrl group. However, the levels of most upregulated metabolites in the Pal + DPA-EA group were remarkably higher than in the Pal and Ctrl groups. Interestingly, DPA-EA administration significantly increased another six SMs and 9 TGs, while Celastrol significantly decreased another 4 SMs and 2 TGs. Different types of DELs and various change trends of the same DELs induced by Celastrol and DPA-EA suggested that they had different other potential mechanisms of action (Figures 6, 7).

**FIGURE 8:**
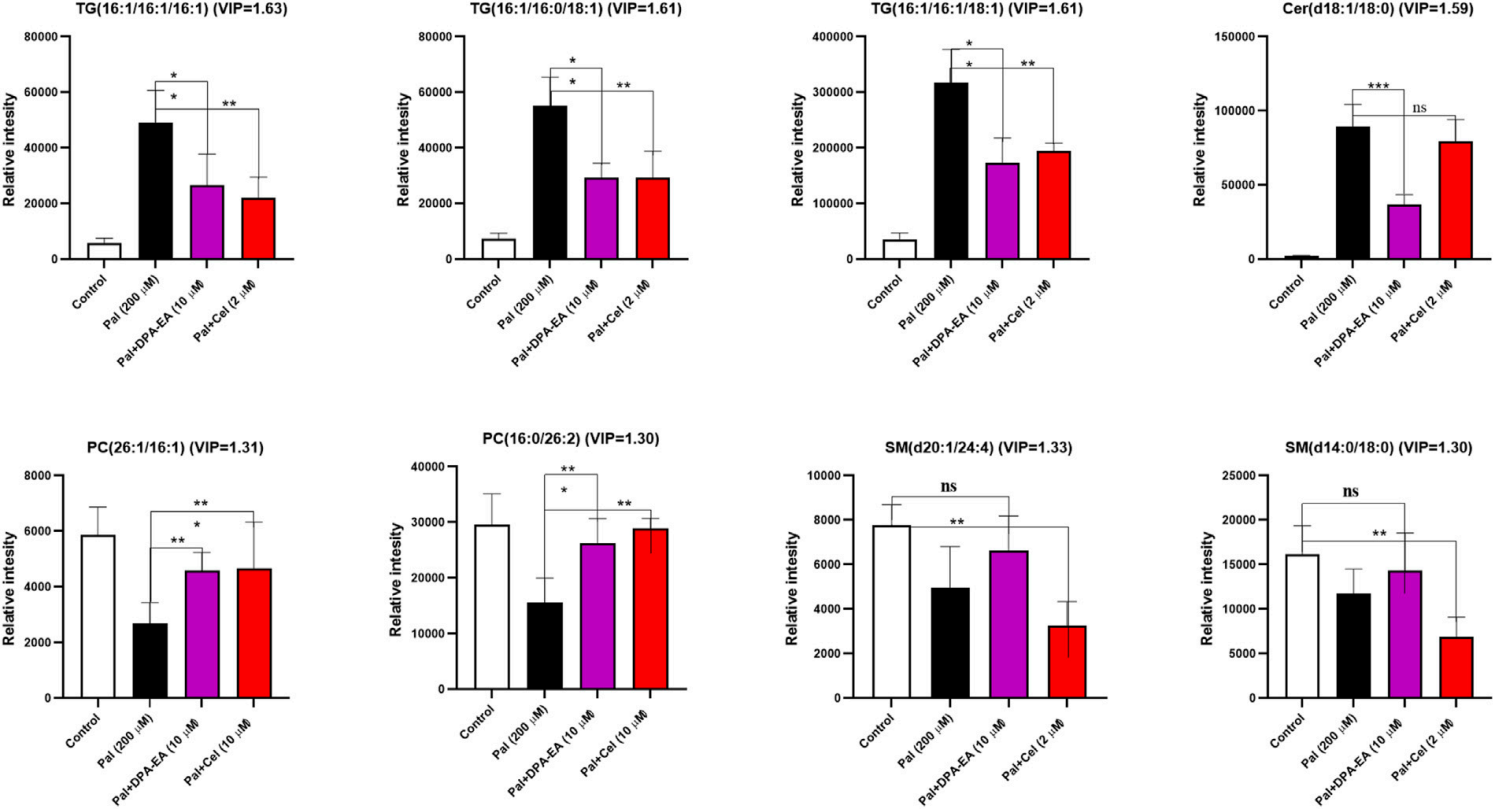
Several potential lipid biomarkers (FC ≥ 2 or ≤0.5; VIP ≥1) in response to the effects of DPA-EA and Celastrol on lipid metabolism. All data in the figure are shown as mean ± SD (n ≥ 3). Bars represent the standard deviation from triplicate determinations. Student’s t-test was used for the statistical analysis, *p < 0.05 and **p < 0.01.

Finally, several typical lipid metabolites were selected for further investigation. As depicted in Figure 8, trend clusters of eight significantly changed lipids were noted for comparative analysis. DPA-EA and Celastrol mainly reversed the Pal-induced upregulation of TG lipids, in which TG (16:1/16:1/16:1), TG (16:1/16:0/18:1), and TG (16:1/16:1/18:1) ranked as the three most significant lipid metabolites. Meanwhile, as for Pal-induced downregulated lipid metabolites, both DPA-EA and Celastrol significantly upregulated PCs, in which PC (26:1/16:1) and PC (16:0/26:2) were the two representative metabolites. These overlapped metabolites might be associated with their common target, Nur77. It was worth noting that DPA-EA and Celastrol also had different effects on lipidomics profile in Pal-induced fatty liver HepG2 cells. For example, DPA-EA significantly downregulated the expression of Cer (d18:1/18:0), but Celastrol had no significant effect on it. Also, we found that DPA-EA remarkably elevated sphingomyelin expressions such as Sph (d18:1) and SM (d16:2/24:4). However, Celastrol did not affect the levels of these two lipid metabolites. It might be attributed to their different additional targets.

### 3.3 The DPA-EA and DHA-EA mixture has a significant anti-inflammatory and anti-obesity effects with excellent safety properties

Our previous work demonstrated that ω-3 PUFA-EA derivatives such as DPA-EA, DHA-EA, and EPA-EA had excellent anti-inflammatory effects acting as Nur77 modulators (Fang et al., 2020). In the pre-part of this work, we further confirmed that the represented compound DPA-EA exhibited excellent lipid-lowering activities with lower toxicity than Celastrol. Moreover, lipidomics analysis revealed that DPA-EA could remodel lipid profile and improve lipid metabolism in the Pal-simulated HepG2 cell model. It suggested that anti-inflammatory Nur77-targeting compounds, DPA-EA and Celastrol, alleviated lipid accumulation by promoting the hydrolysis of PEs and TGs. Based on these findings, we hypothesized the algae oil-derived PUFA-EA mixture (the DPA-EA and DHA-EA mixture) might also exert excellent anti-obesity effects like Celastrol but with low toxicity. Therefore, algal oil containing DHA (16.23%) and DPA (5.22%) was first reacted with ethanolamine without any solvents at 80°C for 24 h and then purified on a silica gel column to obtain the PUFA-EA mixture of DHA-EA (67.54%) and DPA-EA (27.96%) (Supplementary Scheme S1 and Supplementary Figure S1).

#### 3.3.1 The anti-inflammatory activity of the PUFA-EA mixture

**FIGURE 9:**
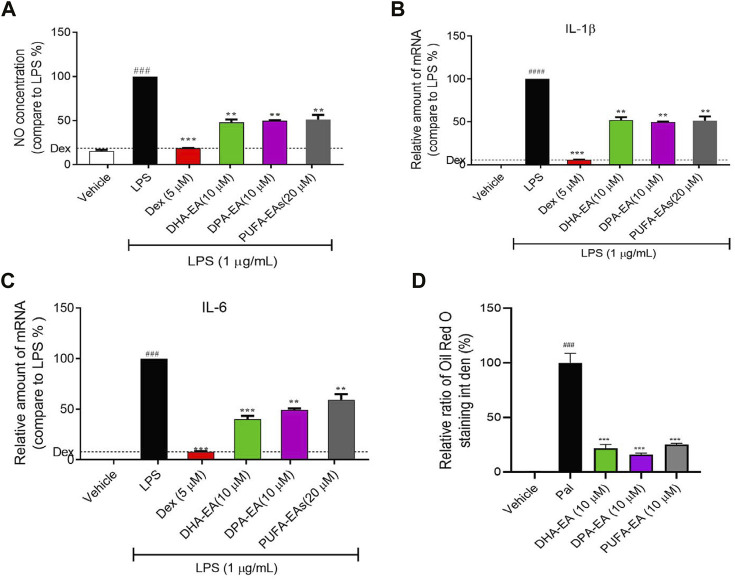
The PUFA-EA mixture showed excellent anti-inflammation activities in LPS-stimulated RAW264.7 cells. **(A)** The NO levels in the medium. **(B,C)** The mRNA levels of IL-1β and IL-6. **(D)** Quantification analysis of Oil Red O staining in Supplementary Figure S7. Int den stands for integrated density. All data in the figure are shown as mean ± SD (n ≥ 3). Bars represent the standard deviation from triplicate determinations. Student’s t-test was used for the statistical analysis, #*p* < 0.05, ##*p* < 0.01, and ###*p* < 0.001, compared with the control group; *p < 0.05 and **p < 0.01, ***p < 0.001, compared with the LPS alone-stimulated group.

First, the synthesized PUFA-EA mixture was further evaluated for *in vitro* anti-inflammatory activity. As shown in Figures 9A–D, LPS treatment caused a dramatic increase in NO release and the expressions of pro-inflammatory cytokines IL-1β and IL-6 compared to control, while DPA-EA, DHA-EA, and the PUFA-EA mixture could significantly alleviate LPS-induced NO release and pro-inflammatory cytokine expressions at the concentration of 10 μM (Figures 9A–C). Also, the PUFA-EA mixture significantly reduced the protein expression of TNF-α and IL-1β induced by LPS in a dose-dependent manner (Supplementary Figure S6). These results implied that the PUFA-EA mixture exhibited anti-inflammatory activity like their monomer molecules (Figures 9A–C and Supplementary Figure S6). In addition, Oil red O staining further demonstrated that PUFA-EA had similar lipid-lowering activity with DHA-EA and DPA-EA (Figure 9D and Supplementary Figure S7).

#### 3.3.2 The anti-obesity activity of PUFA-EAs in HFD-fed obesity mice model

**FIGURE 10:**
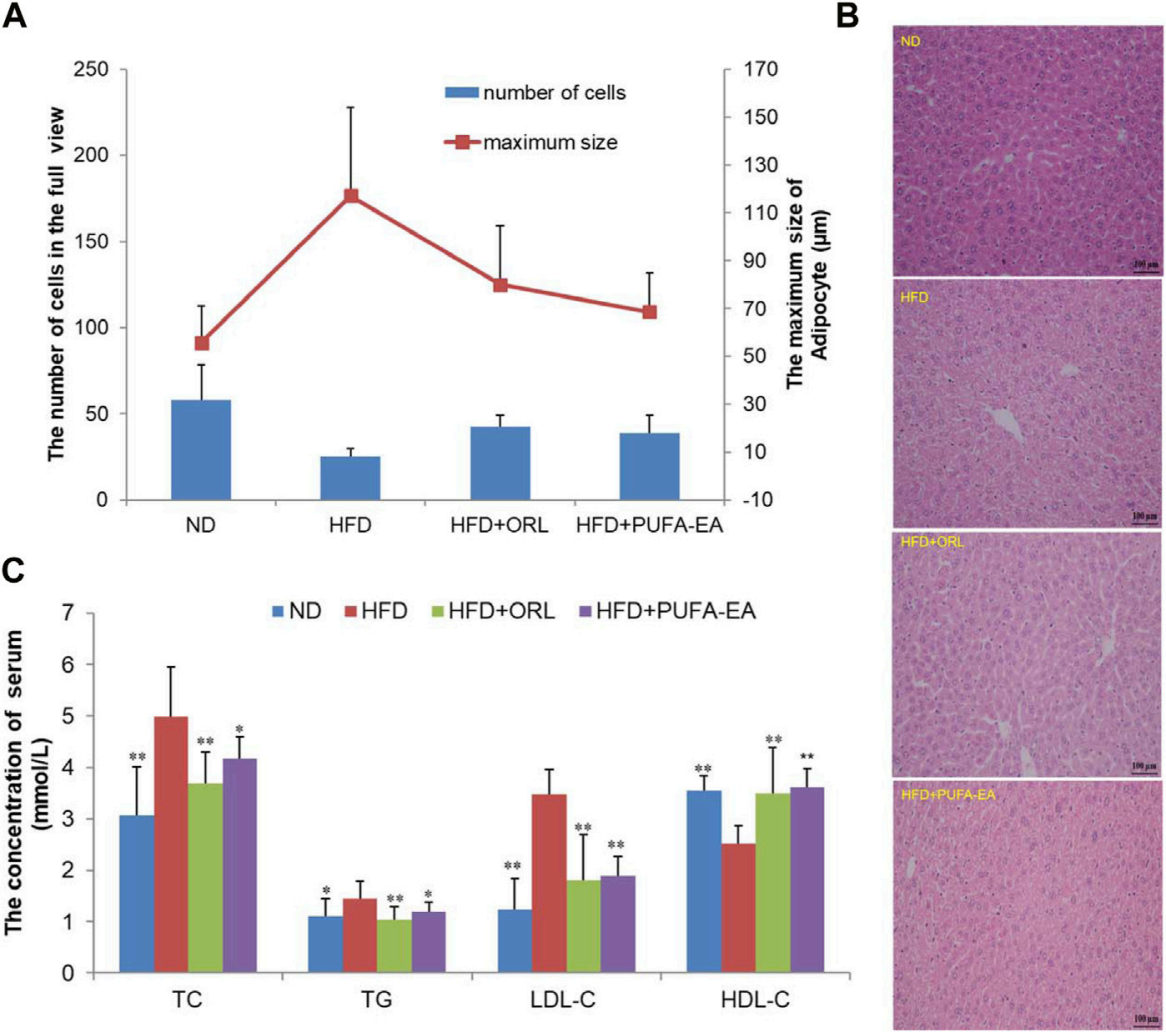
The PUFA-EA mixture showed *in vivo* anti-obesity effects in HFD mouse models. **(A)** The maximum size of adipocyte and the number of cells of the experimental mice’s liver tissue in the full view captured with the microscope (200×). All data in the figure are shown as mean ± SD. Bars represent the standard deviation from duplicate determinations. **(B)** H&E staining of the liver after PUFA-EA supplementation. **(C)** Effects of PUFA-EA on plasma biochemical indicators (TC, TG, HDL-C, and LDL-C) in different groups at the end of 6-week feeding (n = 10). All data in the figure are shown as mean ± SD. Bars represent the standard deviation from duplicate determinations. *p < 0.05 and **p < 0.01.

Next, the algae oil-derived PUFA-EA mixture was considered for the *in vivo* anti-obesity activity in the HFD-fed obesity mice model, with a well-known anti-obesity drug, Orlistat, as the positive control. The experiment results are summarized in Table 2 and Figure 10A–C.

**TABLE 2:** The effects of Orlistat and the PUFA-EA mixture on body weight gain, food intake, and tissue weights in HFD-fed obese mice at the end of 6-week feeding (n = 10)^a^.

	ND	HFD	HFD + ORL	HFD + PUFA-EA
Body weight				
*Initial body weight (g)	21.96 ± 1.34	21.92 ± 1.14	21.49 ± 1.49	21.73 ± 1.04
*Final body weight (g)	31.74 ± 4.06	34.68 ± 3.58	29.29 ± 4.26**	29.30 ± 2.39**
*Net weight gain (g)	9.78 ± 3.17	12.76 ± 3.44	7.80 ± 4.42*	7.57 ± 3.09**
Total food intake (g/d)	7.31 ± 0.87**	8.35 ± 1.16	7.55 ± 1.04**	7.27 ± 0.97**
PER^b^	3.72 ± 1.20*	4.24 ± 1.14	2.87 ± 1.63**	2.89 ± 1.18**
Body length				
*Initial body length (cm)	7.85 ± 0.20	7.77 ± 0.18	7.81 ± 0.19	7.87 ± 0.20
*Final body length (cm)	9.68 ± 0.56	10.02 ± 0.58	9.32 ± 0.62*	9.52 ± 0.25*
Lee’s index^c^	14.83 ± 0.43	15.11 ± 0.28	14.62 ± 0.59*	14.54 ± 0.36**
Liver weight (g)	1.52 ± 0.29**	1.92 ± 0.21	1.48 ± 0.18**	1.46 ± 0.18**
Liver index (%)^d^	4.79 ± 0.58**	5.55 ± 0.57	5.11 ± 0.60**	4.97 ± 0.29**
White adipose tissues (WAT)				
*Epididymal fat (g)	0.52 ± 0.11**	0.84 ± 0.21	0.50 ± 0.18**	0.47 ± 0.10**
*Perirenal fat (g)	0.31 ± 0.09 **	0.55 ± 0.10	0.31 ± 0.15*	0.35 ± 0.12**
Fat coefficient (%)^e^	2.62 ± 0.27**	4.02 ± 1.20	2.70 ± 0.87*	2.80 ± 0.29**

^a^ All data in the table are shown as mean ± SD. *p < 0.05, **p < 0.01 vs*.* the HFD, group.

^b^ PER, food efficiency ratio (%) = [body weight gain (g)/diet consumed (g)] × 100.

^c^ Lee’s index = [body weight (g) × 1000/body length (cm)]^1/3^.

^d^ Liver index (%) = liver weight (g)/body weight (g) × 100.

^e^ Fat coefficient (%) = [epididymal fat (g) + perirenal fat (g)]/body weight (g) × 100.

##### 3.3.2.1 The effects of PUFA-EA mixture on the body weight, food intake, liver weight, and white adipose tissue

As shown in Table 2, The initial body weights of experimental mice were similar (*p* > 0.05), and the body weight increased significantly at the end of the obesity-modeling period (6 weeks). Compared to the initial body weight, the final body weight of the ND, HFD, HFD + ORL, and HFD + PUFA-EAs groups increased by 9.78 ± 3.17 g (44.54%), 12.76 ± 3.44 g (58.21%), 7.80 ± 4.42 g (36.30%), and 7.57 ± 3.09 g (34.84%), respectively. The net body-weight gain, total food intake, liver index, and fat coefficient of the HFD group were significantly higher than those of the ND group (*p* < 0.01), indicating that HFD intake promoted the development of obesity in ICR mice. However, compared to the HFD group, The HFD + ORL group showed significantly lower values of final body weight, PER, Lee’s index, liver index, and WAT weight. It indicated that the Orlistat administration could reverse these changes upon the HFD challenge. A similar inhibitory effect was also observed in the PUFA-EAs group. For example, the final body weights of the HFD + ORL and HFD + PUFA-EAs groups were significantly lower than those of the HFD group (*p* < 0.01). The fat coefficient of the HFD + ORL and HFD + PUFA-EA groups were also significantly lower than those of the HFD group (*p* < 0.05). Besides, the inhibitory effect of Orlistat and PUFA-EAs on food intake was no significant difference. These results implied that PUFA-EAs had good fat-reducing potential in obese mice, equivalent to Orlistat (*p* > 0.05).

##### 3.3.2.2 The effects of PUFA-EAs on the pathology of the liver in mice

The morphological structure of the experimental mice’s liver tissues was observed using a microscope. The maximum size of adipocytes and the calculated number of liver cells in the full view captured with the microscope (200×) were shown in Figure 10A. Compared with the ND group, fewer cells in the full view were calculated in the HFD group, while cell numbers could be increased after the treatment of Orlistat and the PUFA-EA mixture. Therefore, larger adipocytes were observed in the HFD group, which could be inhibited by the supplementations of Orlistat and the PUFA-EA mixture; Figure 10B shows the H&E staining pathology of mice’s liver tissue. The hepatic tissue sections in the ND group showed normal hepatocytes arranged neatly. However, apparent lipid droplets diffusely appeared in the livers of HFD-fed mice, and the boundary between individual liver cells is blurred compared with that of the ND group. The results are similar as reported previously (Tsuru et al., 2020). The PUFA-EA mixture, like Orlistat, effectively reduced the number and volume of lipid droplets induced by the HFD and clarified the cell boundaries. These findings suggested that the administration of the algae oil-derived PUFA-EA mixture produced an excellent inhibitory effect on obesity caused by an HFD.

##### 3.3.2.3 Determination of the blood lipid-related indices (HDL- C, LDL- C, TC, and TG)

Feeding an HFD for 6 weeks could cause obesity, hyperlipidemia, and hyperglycemia in ICR mice (Li J. L. et al., 2020). It is a good model for studying diet-induced obesity. To further confirm the *in vivo* lipid-lowering effects of algae oil-derived PUFA-EA mixture, we measured the changes of crucial blood biochemical indexes upon HFD challenge and drug administration. Values of lipid parameters at the end of the experiment were presented in Figure 10C. The serum TC, TG, and LDL-C levels in the HFD group were significantly increased by 63.07%, 30.63%, and 182.93%, respectively, compared to those of the control group (*p* < 0.01). Meanwhile, HDL-C levels decreased by 40.87% in the HFD group compared to the control group (*p* < 0.01). However, the treatment of the PUFA-EA mixture could significantly inhibit the increased levels of TG, TC, and LDL-C induced by HFD, with inhibition rates of 21.85%, 19.38%, and 84.12%, respectively. At the same time, the treatment of the PUFA-EA mixture significantly increased the HDL-C level (upon (%), 43.65) relative to HFD-fed mice. A reduced ratio of LDL-C/HDL-C was also observed in the PUFA-EA and Orlistat groups’ mice. The effect of the PUFA-EA mixture on the blood lipid-related indices is similar to that of Orlistat treatment, implying the potent anti-obesity activity *in vivo*.

#### 3.3.3 The acute toxicity study of the PUFA-EA mixture in mice

To verify the *in vivo* safety of the algae oil-derived PUFA-EA mixture, we evaluated the acute toxicity in mice. The daily dose of 54,000 mg/kg of PUFA-EA mixture did not lead to any mortality throughout the study period of 14 days. All parameters observed were normal even when the limit dose was maintained at 54,000 mg/kg body weight (Supplementary Table S7). The autopsy results of all mice at the end of the experimental period (14 days) also revealed that no apparent changes were observed in any mice organs. These results indicated that the algae oil-derived PUFA-EA mixture had no acute toxicity in mice at a dose of 54,000 mg/kg (significantly exceeding the limit dose of 5,000 mg/kg/day), and the PUFA-EA mixture at a higher dose is unnecessary. The algae oil-derived PUFA-EAs was practically non-toxic *in vivo*, indicating the great therapeutic and safety potential for further development as an anti-obesity agent.

## 4 Discussion

**FIGURE 11:**
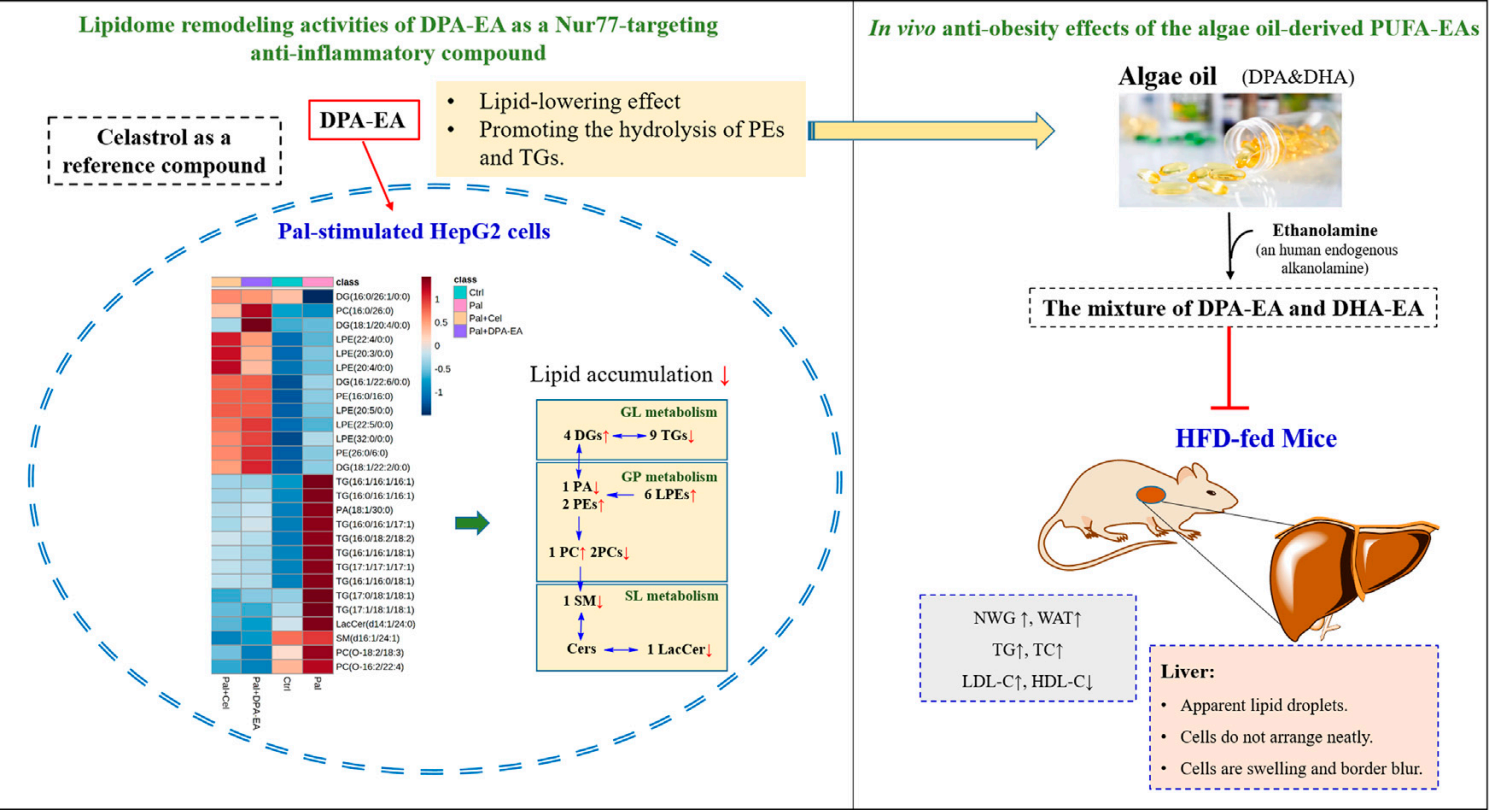
Lipidome remodeling activities of DPA-EA by targeting Nur77 and in vivo anti-obesity effects of the algae oil-derived PUFA-EAs.

Nur77 plays a crucial role in the inflammation process and lipid metabolism. Moreover, Nur77-targeting anti-inflammatory compounds are a potential therapeutic strategy for treating inflammation-related diseases such as obesity. However, the lipid-lowering mechanism of anti-inflammatory compounds targeting Nur77 remains unclear and has not been investigated. Understanding lipid profile changes induced by specific Nur77 modulators in the lipid-lowering process is crucial to exploring the underlying mechanism of their anti-obesity effects. The reference compound, Celastrol, has been proven to Nur77-dependently inhibit chronic inflammation and play a role in weight loss in obese animals (Wang et al., 2011). DHA, an essential ω-3 PUFA with anti-inflammatory and anti-obesity activities (Wei et al., 2021), is recently found to be an endogenous lipid reported to bind to Nur77^21^. Additionally, our previous work demonstrated that ω-3 PUFA-EA derivatives such as DPA-EA displayed excellent anti-inflammatory effects in a Nur77-dependent manner^21^. NAFLD is the most common liver disease associated directly with obesity. The present work also demonstrated that DPA-EA and Celastrol had excellent lipid-lowering lipid profile-remodeling activities in the liver HepG2 Cell Model (Figure 11).

Pal is a saturated fatty acid that has been reported to induce pro-inflammatory production in hepatocytes. The overload treatment of Pal causes triglyceride accumulation into specialized organelles termed lipid droplets. When rendering lipid droplets and hepatic lipotoxicity, Pal can cause glucotoxicity, oxidative stress, apoptosis, and endoplasmic reticulum (ER) and mitochondrial dysfunction in HepG2 cells (Zang et al., 2018; Eynaudi et al., 2021). The present work also demonstrated that Pal administration caused lipid accumulation in HepG2 cells, with a significant elevation of lipid droplets. Besides, several lipidomics studies have noted that Pal can influence the overall lipid metabolism (Shih et al., 2018; Cabezas et al., 2022). For example, Pal insult could affect lipid metabolites such as PE, PS, PC, and glycerophosphocholine (GPC) (Cabezas et al., 2022). The stable isotope-labeled lipidomics on Pal-stimulated HepG2 cells revealed that Pal significantly altered the levels of many lipids (*e.g.,* PL, Cer, GL, *et al.*), which were involved in biosynthetic pathways of DG, TG, PI, dihydroceramide (dHCer), Cer, SM, PC, PE, LPA, and LPE (Shih et al., 2018). Our lipidomics study also showed Pal-induced a significantly dysregulated lipid profile in HepG2 cells. We found 17 types of DELs between the control and Pal groups, mainly Cer, PC, TG, PE, Sph, SM, *etc.* Among them, most metabolites of glycerides (GLs), GPs, and sphingomyelin were significantly upregulated by Pal, implying that Pal treatment led to severe lipid gathering in HepG2 cells.

TGs are the primary storage and transport form of fatty acids in cells and plasma (Alves-Bezerra and Cohen, 2017). Lipid accumulation (*e.g.*, TG accumulation) in NAFLD has been reported to be one of the causes of insulin resistance in the liver (Gaggini et al., 2013). Moreover, the progressive increases in TG content are associated with progressive impairment of insulin action in the liver, skeletal muscle, and adipose tissue in non-diabetic obese subjects (Korenblat et al., 2008). Reducing triglyceride content *via* triglyceride synthase DAGT2 inhibition can improve hepatic steatosis in obese mice with non-alcoholic steatohepatitis (Yamaguchi et al., 2007). In our data, TGs involved in the insulin resistance transduction pathway, such as TAG (C57:1), TAG (C48:1), and TAG (C54:5) (Al-Sulaiti et al., 2018), were also identified as differential metabolites between the Ctrl and Pal groups, presented as TG (12:0/14:0/22:1) and TG (16:0/16:0/22:5). Additionally, DPA-EA and Celastrol could reverse the Pal-induced elevation of TGs in HepG2 cells, especially TG (16:1/16:1/16:1), TG (16:0/16:1/16:1), TG (17:1/17:1/17:1) *et al.* On the other hand, MGs and DGs are precursor molecules for the synthesis of TG. Diglycerides (DGs), with essential functions in lowering blood lipids, reducing visceral fat, and inhibiting weight gain (Hue et al., 2009), are mainly produced by blocking the accumulation of TG in the body. In Pal-stimulated HepG2 cells, both DPA-EA and Celastrol markedly upregulated the contents of several DGs, particularly DG (16:1/22:6/0:0), DG (18:1/22:2/0:0), DG (18:1/20:4/0:0) and DG (16:0/26:1/0:0). Thus, DPA-EA and Celastrol might alleviate lipid accumulation by promoting the hydrolysis of triglycerides or lower triglyceride expression. In all, DPA-EA and Celastrol robustly affected GL metabolism and metabolites of GL species (TG and DG) in Pal-induced HepG2 cells.

GPs (*e.g.*, PC, PE, PG, *et al.*) regulate lipid metabolism, lipoprotein, and whole-body energy metabolism, too (van der Veen et al., 2017). PCs are the most abundant phospholipids in all mammalian cell membranes (van der Veen et al., 2017). In the present study, GP metabolism was significantly altered by DPA-EA and Celastrol in Pal-induced HepG2 cells. It is reported that PC therapy can relieve HFD-induced obesity and obesity-related complications by reducing TG and TC levels in serum (Lee et al., 2014). Buang *et al.* also reported that dietary PC could attenuate orotate-induced fatty liver in Sprague-Dawley rats by increasing CPT1 activity and preventing TG accumulation (Kumar et al., 2021). However, it was also found that some PCs were increased in the LPS-stimulated zebrafish model and acute TNFα-treated HepG2 cells (Chico et al., 2019; Luo et al., 2019). These data suggests various PCs may have different functions in the progress of inflammation and NAFLD. Interestingly, our results showed that in Pal-stimulated HepG2 cells, DPA-EA and Celastrol could significantly decrease PC(O-16:2/22:4) and PC(O-18:2/18:3) and elevated PC (16:0/26:0) or other PCs (8 for Celastrol and seven for DPA-EA). PEs are the second most abundant GPs in eukaryotic cells. Clinical research shows that a standard PC/PE ratio (∼1.5–1.8) is relevant to liver health and a large proportion of NAFLD patients have abnormally low cellular PC/PE ratios (van der Veen et al., 2017). Besides, it is reported that PEs are significantly lower in human fatty livers (Puri et al., 2007). Notably, our present work exhibited that DPA-EA and Celastrol administrations caused a steady increase of two typical PEs, PE (16:0/16:0) and PE (26:0/6:0), in Pal-induced HepG2 cells. Celastrol also elevated PE (P-16:0/14:0) (P-18:0/20:0), and PE (O-18:2/19:0), while DPA-EA increased PE (6:0/16:0) (10:0/16:0), PE (16:1/20:3), and PE (14:0/14:0). However, Celastrol downregulated most of the other PEs but DPA-EA did not. Together, different classes of GPs displayed various alterations due to DPA-EA and Celastrol perturbation. PCs and PEs might not be the lipid markers to illustrate the underlying anti-inflammation and anti-obesity mechanism that Nur77-targeting anti-inflammatory compounds mediated.

Lysophospholipids (*e.g.*, LPCs and LPEs) are signaling molecules that play an essential role in inflammation, insulin resistance, and fatty liver disease (Zu and Cham, 2008). The interconversion between phospholipids and lysophospholipids is catalyzed by phospholipase A2 (D'Arrigo and Servi, 2010). Many studies show that LPC and LPE are reduced due to obesity or obesity-associated insulin resistance (Barber et al., 2012; Weir et al., 2013; Heimerl et al., 2014). For example, the circulating concentrations of some plasma LPEs (*e.g.*, LPE (14:1), LPE (18:1), and LPE (18:2)) are lower in obese subjects than in normal-weight subjects (Del Bas et al., 2016). Interestingly, our results showed that both DPA-EA and Celastrol could slightly increase the content of LPEs, including LPE (20:4/0:0), LPE (22:4/0:0), LPE (20:3/0:0), LPE (20:5/0:0), LPE (22:5/0:0), and LPE (32:0/0:0). It is consistent with the proposal that LPE plays a vital role in obesity and inflammation. Additionally, MN Barber *et al.* reported that levels of LPCs in the plasma of HFD-fed mice were stably decreased (Barber et al., 2012; Heimerl et al., 2014). As expected, both DPA-EA and Celastrol could upregulate the levels of LPCs in Pal-induced HepG2 cells. However, Celastrol upregulated LPC more significantly than DPA-EA. Celastrol increased another nine LPCs, such as LPC (22:6/0:0), LPC (20:5/0:0), and LPC (22:2/0:0). In comparison, DPA-EA enhanced the expression of another three LPCs including LPC (20:0/0:0), LPC (18:0/0:0), and LPC (O-22:0/0:0). Whatever, both DPA-EA and Celastrol significantly upregulated the expressions of LPE and LPC species.

Sphingolipids such as Cer and SM are also associated with the progression of obesity and insulin resistance (Haus et al., 2009; Sokolowska and Blachnio-Zabielska, 2019). Cer levels are increased in insulin-resistant individuals (Adams et al., 2004). The inhibition of Cer synthesis can improve glucocorticoid, saturated fat, and obesity-induced insulin resistance (Holland et al., 2007). An unbalanced ratio between ceramides and terminal metabolic products in the liver and plasma promotes weight gain, inflammation, and insulin resistance (Regnier et al., 2019). Similarly, we found that Pal could increase Cer in HepG2 cells. Both DPA-EA and Celastrol significantly reversed the Pal-induced increase of LacCer(d14:1/24:0). Especially, DPA-EA also diminished the Pal-induced increase of several other Cers, including GlcCer(d18:1/22:1), GlcCer(d18:1/22:0), Cer(d18:1/20:0), and LacCer(d14:0/24:0), but Celastrol did not. At the same time, in Pal-induced HepG2 cells, DPA-EA markedly upregulated another 11 Cers, but Celastrol significantly increased no Cer. Additionally, Jorge Simon *et al.* reported that the levels of sphingomyelin and ceramide were elevated during steatosis and non-alcoholic steatohepatitis (NASH) (Simon et al., 2019). Our present study showed that DPA-EA and Celastrol could significantly co-decrease the Pal-upregulated expression of SM (d16:1/24:1). Celastrol also downregulated the expression levels of SM (d14:2/21:0) and SM (d14:1/19:0) but upregulated no other SM. DPA-EA could not downregulate other SMs but significantly upregulate the contents of SM (d14:1/20:0), SM (d14:2/24:4), SM (d16:2/24:4), SM (d16:1/24:4), SM (d15:1/20:1), SM (d15:1/27:0), SM (d18:2/26:1), and Sph (d18:1). Based on those above-mentioned, it was concluded that DPA-EA is more sensitive to Cer than Celastrol, but Celastrol might cause over-downregulation of SM. Interestingly, it was reported that Celastrol decreased the levels of SM and Sph in the blood when inducing liver injury in mice (Zhang et al., 2019). Thus, the over-downregulation of SM may be associated with the off-target toxicity of Celastrol.

Nur77 is a crucial physiological modulator of lipid metabolism in adipose tissue. Female Nur77-deficiency mice show decreased lipolysis in white adipose tissue and increased hepatic fat storage (Perez-Sieira et al., 2013). The liver tissue of Nur77-null mice can accumulate more TGs than wild-type mice (Chao et al., 2009). However, adenovirus-mediated Nur77 overexpression reduces hepatic triglyceride levels while increasing plasma LDL-C and decreasing HDL-C (Hu et al., 2014). In live HepG2 cells, Pal can induce the downregulation of Nur77 and lipid accumulation, while Nur77 overexpression can improve the phenomenon of lipotoxicity (Zhao et al., 2018). Additionally, Celastrol can Nur77-dependently inhibit chronic inflammation in obese mice, thereby protecting them against HFD-induced obesity (decreasing body weight, adipose tissue mass, and the size of adipocytes and ameliorating HFD-caused fatty liver). Excitedly, the present work demonstrated that both DPA-EA and Celastrol displayed lipid-lowering activities in Pal-stimulated HepG2 cells. Using lipidomics analysis, we found that DPA-EA and Celastrol could co-downregulate the TG expressions but upregulate DG expressions in Pal-stimulated HepG2 cells. It is consistent with the effects of Nur77 overexpression (Zhao et al., 2018). In particular, 27 lipid metabolites with statistically significant changes were identified as critical factors responding to the lipid-lowering process of DPA-EA and Celastrol (Figure 11).

In summary, the present study demonstrates that anti-inflammatory Nur77 modulators can decrease lipid accumulation by promoting lipolysis. Moreover, a total of 27 lipids were identified as potential biomarkers that responded to the anti-obesity effect of Nur77-targeting anti-inflammatory compounds. These biomarkers have important implications for developing anti-obesity agents targeting Nur77 and predicting the recovery of aberrant lipid metabolism in obesity-related metabolic disorders. Notably, the algae oil-derived PUFA-EA mixture showed excellent anti-obesity effects on HFD-fed mice (Figure 11) with practically no toxicity *in vivo*. Overall, we provide new insights into the intervention mechanism of Nur77-targeting anti-inflammatory compounds on lipid metabolism. In particular, the algae oil-derived PUFA-EA mixture may be a promising therapeutic agent of obesity and NAFLD worthy of further development.

## Data Availability

The original contributions presented in the study are included in the article/Supplementary Material, further inquiries can be directed to the corresponding author.
