# Supplementary material for: Lipidome remodeling activities of DPA-EA in palmitic acid-stimulated HepG2 cells and the in vivo anti-obesity effect of the DPA-EA and DHA-EA mixture prepared from algae oil
Source: Front Pharmacol. 2023 Mar 29;14:1146276. doi: 10.3389/fphar.2023.1146276 (PMC10090563; doi:10.3389/fphar.2023.1146276)
Supplement: Supplementary file 1 [file Table1.DOCX]

**SUPPORTING INFORMATION**

**Lipidome Remodeling Activities of DPA-EA in Palmitic Acid-Stimulated HepG2 Cells and The in vivo Anti-Obesity Effect of the DPA-EA and DHA-EA Mixture Prepared from Algae Oil**

Hua Fang ^a, c, #^, Yin Cao^b, #^, JianYu Zhang^b^, Xiumei Wang^b^, Mengyu Li^a,c^, Zhuan Hong^a^, Zhen Wu^b^, Meijuan Fang^b*^

^a^ Technical Innovation Center for Utilization of Marine Biological Resources, Ministry of Natural Resources, Xiamen, 361005, China

^b^ Fujian Provincial Key Laboratory of Innovative Drug Target Research and State Key Laboratory of Cellular Stress Biology, School of Pharmaceutical Sciences, Xiamen University, Xiamen 361102, China

^c^ College of Food and Biological Engineering, Jimei University, Xiamen 361021, China

^#^ These authors contribute equally to this work.

^*^ Corresponding author, Meijuan Fang: orcid.org/0000-0002-3205-9037; Tel.: +86-592-2189868; Fax: +86-592-2189868; E-mail addresses: fangmj@xmu.edu.cn.

| **Scheme S1**  **Table S1**  **Table S2**  **Table S3**  **Table S4**  **Table S5**  **Table S6**  **Table S7**  **Figure S1**  **Figure S2**  **Figure S3**  **Figure S4**  **Figure S5**  **Figure S6**  **Figure S7**  **Figure S8** | S3  S4  S5  S6  S7  S8  S9  S10  S11  S12  S13  S14  S15  S16  S17  S18 |
| --- | --- |

**Scheme S1.** Preparation of the algae oil-derived PUFA-EAs

**Table S1**. Corresponding values of R^2^X and Q^2^ of PCA models

| No. | Description | ion mode | N | R^2^X(cum) | Q^2^(cum) |
| --- | --- | --- | --- | --- | --- |
| 1 | Ctrl, Pal, Pal+DPA-EA, and Pal+Cel | (+) | 28 | 0.775 | 0.695 |
|  |  | (-) | 28 | 0.783 | 0.677 |
| 2 | Pal *vs.* Ctrl | (+) | 14 | 0.745 | 0.661 |
|  |  | (-) | 14 | 0.766 | 0.671 |
| 3 | Pal+Cel *vs.* Pal | (+) | 14 | 0.673 | 0.519 |
|  |  | (-) | 14 | 0.689 | 0.562 |
| 4 | Pal+DPA-EA *vs.* Pal | (+) | 14 | 0.63 | 0.477 |
|  |  | (-) | 14 | 0.66 | 0.498 |

Ctrl: Control (DMSO) group; Pal: Pal-stimulated HepG2 group; Pal+Cel: Pal-stimulated HepG2 cells with celastrol administration; Pal+DPA-EA: Pal-stimulated HepG2 cells with DPA-EA intervention.

**Table S2**. Classifications and profile of differentially expressed lipids (DELs) regulated among each group.

| Groups | | Ctrl *vs.* Pal | | PA *vs.* Pal+Cel | | PA *vs.* Pal+DPA-EA | |
| --- | --- | --- | --- | --- | --- | --- | --- |
| Lipid Class | ion mode | Pos (+) | Neg (-) | Pos (+) | Neg (-) | Pos (+) | Neg (-) |
| GL | TG | 138 |  | 92 (82) |  | 106 (85) |  |
|  | DG | 23 |  | 27 (15) |  | 20 (14) |  |
|  | MG | 3 |  | 3 (1) |  | 3 (1) |  |
| CE | CE | 9 |  | 6 (4) |  | 8 (5) |  |
| GP | PC | 155 | 77 | 119 (79) | 71 (43) | 137 (93) | 83 (48) |
|  | PE | 48 | 72 | 56 (38) | 61 (34) | 41 (27) | 45 (21) |
|  | PA | 1 | 16 | 1 (1) | 15 (11) | 1 (1) | 12 (7) |
|  | PI | 25 | 33 | 28 (16) | 37 (16) | 6 (5) | 32 (15) |
|  | PS | 35 | 23 | 27 (16) | 22 (14) | 24 (15) | 17 (10) |
|  | PG | 9 | 59 | 8 (6) | 48 (35) | 7 (7) | 53 (33) |
| lysoGP | LPC | 27 | 24 | 22 (21) | 15 (15) | 9 (8) | 6 (5) |
|  | LPE | 9 | 15 | 7 (7) | 12 (11) | 10 (9) | 14 (12) |
|  | LPI | 1 | 6 |  |  |  |  |
|  | LPS | 1 | 1 |  | 2 (0) | 1 (1) | 1 (0) |
|  | LPA |  | 1 |  |  |  | 1 (1) |
|  | LPG |  | 1 |  |  |  | 1 (0) |
| Cer | Cer | 32 | 11 | 14 (10) | 9 (5) | 21 (18) | 11 (8) |
|  | GlcCer | 17 |  | 7 (6) |  | 12 (10) |  |
|  | LacCer | 3 | 3 | 4 (2) |  | 4 (2) | 9 (3) |
| Sphingomyelin | SM | 34 | 21 | 48 (31) | 34 (17) | 33 (19) | 29 (14) |
|  | Sph | 1 |  |  |  | 1 (1) |  |
| Total |  | 571 | 363 | 469 (335) | 326 (201) | 444 (321) | 314 (177) |
| Total Differential lipids |  | 865^*^ | | 722^*^ (480) | | 705^*^ (457) | |
| Co-regulated lipids |  |  |  | 443 DELs co-regulated by Cel and DPA-EA | | | |

Numbers of DELs in each lipid classifications are listed and numbers in brackets indicated DELs between these two group that are common with DELs regulated in model group (Pal group *versus* Ctrl group). Empty data indicated no corresponding lipid substances selected.

^*^ : Number = lipids in positive mode + lipids in negative mode – common lipids in both positive and negative.

**Table S3.** Classifications and profile of DELs in each group.

| Groups | | Pal *vs.* Ctrl | | Pal+Cel *vs.* Pal | | Pal+DPA-EA *vs.* Pal | |
| --- | --- | --- | --- | --- | --- | --- | --- |
| Lipid Class | | Up | Down | Up | Down | Up | Down |
| GL | TG | 127(103) | 11(2) | 8 | 84(23) | 47(17) | 59(13) |
|  | DG | 15(7) | 8(6) | 27(1) |  | 19(4) | 1 |
|  | MG | 3(2) |  | 3 |  |  | 3(1) |
| CE | CE | 8(1) | 1 |  | 6 |  | 8 |
| GP | PC | 84(34) | 126(36) | 69(2) | 105(11) | 57(10) | 136(11) |
|  | PE | 67(20) | 43(7) | 24(2) | 73(4) | 40(4) | 44(3) |
|  | PA | 17(11) |  | 6(4) | 10(5) | 8(3) | 5(2) |
|  | PI | 38(18) | 18(1) | 3 | 60(11) | 6(2) | 32(1) |
|  | PS | 20(8) | 37(13) | 3 | 45(9) | 8(1) | 32 |
|  | PG | 45(20) | 23(1) | 9(1) | 46(3) | 7(2) | 53(6) |
| lysoGP | LPC | 31(19) | 6 | 15(2) | 12 | 6 | 6 |
|  | LPE | 19(6) |  | 13(2) | 3 | 18(1) | 1 |
|  | LPI | 6(5) |  |  |  |  |  |
|  | LPS | 1(1) |  | 1(1) | 1 | 1 | 1 |
|  | LPA | 1(1) |  |  |  | 1(1) |  |
|  | LPG | 1(1) |  |  |  | 1(1) |  |
| Cer | Cer | 41(32) | 2(1) | 16 | 7 | 24(9) | 8(2) |
|  | GlcCer | 15(13) | 2 |  | 7 | 1 | 11(3) |
|  | LacCer | 5 | 1 | 1 | 3(1) | 2(1) | 10(2) |
| Sphingomyelin | SM | 18(7) | 24(4) | 3 | 59(11) | 14(3) | 34(1) |
|  | Sph | 1(1) |  |  |  | 1(1) |  |
| Total |  | 563(310) | 302(71) | 201(15) | 521(78) | 261(61) | 444(45) |

Numbers of DELs upregulated or down-regulated in each lipid classifications are listed, “up” indicated FC >1 and “down” indicated FC <1. Numbers in brackets indicated DELs FC >2 or FC <0.5. Empty data indicated no corresponding lipid substances selected.

**Table S4.** Classifications and profile of co-regulated DELs by Cel and DPA-EA.

| Groups | | 443 Co-regulated DELs | |
| --- | --- | --- | --- |
| Lipid Class | | Up | Down |
| GL | TG | 7 | 44(9) |
|  | DG | 14(4) |  |
|  | MG |  |  |
| CE | CE |  | 5 |
| GP | PC | 35(1) | 63(2) |
|  | PE | 15(2) | 32 |
|  | PA | 3 | 3(1) |
|  | PI | 1 | 25 |
|  | PS | 2 | 25 |
|  | PG | 1 | 33 |
| lysoGP | LPC | 3 | 3 |
|  | LPE | 11(6) |  |
|  | LPI |  |  |
|  | LPS |  | 1 |
|  | LPA |  |  |
|  | LPG |  |  |
| Cer | Cer | 10 | 2 |
|  | GlcCer |  | 4 |
|  | LacCer | 1 | 2(1) |
| Sphingomyelin | SM | 1 | 30(1) |
|  | Sph |  |  |
| Total |  | 104(13) | 272(14) |

Numbers of DELs upregulated or down-regulated in each lipid classifications are listed, “up” indicated FC >1 and “down” indicated FC <1. Numbers in brackets indicated DELs FC >1.6 or FC <0.5. Empty data indicated no corresponding lipid substances selected.

**Table S5.** Classifications and profile of 279 DELs individually regulated by Cel.

| Groups | | 279 DELs individually regulated by Cel | |
| --- | --- | --- | --- |
| Lipid Class | | Up | Down |
| GL | TG | 1 | 23(2) |
|  | DG | 13(2) |  |
|  | MG |  |  |
| CE | CE |  | 1 |
| GP | PC | 25(7) | 34(7) |
|  | PE | 9(3) | 31(1) |
|  | PA | 3(3) | 5(2) |
|  | PI | 2 | 33(4) |
|  | PS | 1 | 20 |
|  | PG | 5(1) | 11 |
| lysoGP | LPC | 11(4) | 9 |
|  | LPE | 2(1) |  |
|  | LPI |  |  |
|  | LPS | 1(1) |  |
|  | LPA |  |  |
|  | LPG |  |  |
| Cer | Cer | 5 | 5 |
|  | GlcCer |  | 3 |
|  | LacCer |  | 1 |
| Sphingomyelin | SM | 1 | 24(4) |
|  | Sph |  |  |
| Total |  | 79(22) | 200(20) |

Numbers of DELs upregulated or down-regulated in each lipid classifications are listed, “up” indicated FC >1 and “down” indicated FC <1. Numbers in brackets indicated DELs FC >1.6 or FC <0.5. Empty data indicated no corresponding lipid substances selected.

**Table S6.** Classifications and profile of 279 DELs individually regulated by DPA-EA.

| Groups | | 262 DELs individually regulated by DPA-EA | |
| --- | --- | --- | --- |
| Lipid Class | | Up | Down |
| GL | TG | 23(9) | 15(2) |
|  | DG | 5(3) | 1 |
|  | MG |  |  |
| CE | CE |  | 3 |
| GP | PC | 14(8) | 64(3) |
|  | PE | 15(4) | 12 |
|  | PA | 3(2) | 2(1) |
|  | PI | 3(2) | 7(1) |
|  | PS | 6 | 7 |
|  | PG | 4(1) | 17(2) |
| lysoGP | LPC | 3(2) | 2 |
|  | LPE | 4(2) | 1 |
|  | LPI |  |  |
|  | LPS | 1 |  |
|  | LPA | 1(1) |  |
|  | LPG | 1(1) |  |
| Cer | Cer | 14(8) | 5(1) |
|  | GlcCer | 1 | 7(2) |
|  | LacCer | 1(1) | 8(1) |
| Sphingomyelin | SM | 8(6) | 3 |
|  | Sph | 1(1) |  |
| Total |  | 108(51) | 154(13) |

Numbers of DELs upregulated or down-regulated in each lipid classifications are listed, “up” indicated FC >1 and “down” indicated FC <1. Numbers in brackets indicated DELs FC >1.6 or FC <0.5. Empty data indicated no corresponding lipid substances selected.

**Table S7**. Observation on acute toxicity in mice for PUFA-EA

| Item | Control | | Treated by PUFA-EA | |
| --- | --- | --- | --- | --- |
|  | male | Female | male | Female |
| General Appearance | √ | √ | √ | √ |
| Spirit | √ | √ | √ | √ |
| Skin and Fur | √ | √ | √ | √ |
| Eyes, Nose | √ | √ | √ | √ |
| Respiration | √ | √ | √ | √ |
| Urine | Δ | Δ | Δ | Δ |
| Feces | Δ | Δ | Δ | Δ |
| Locomotor | √ | √ | √ | √ |

Note: √ stands for Nommal, and Δ stands for No Discoloration.

**
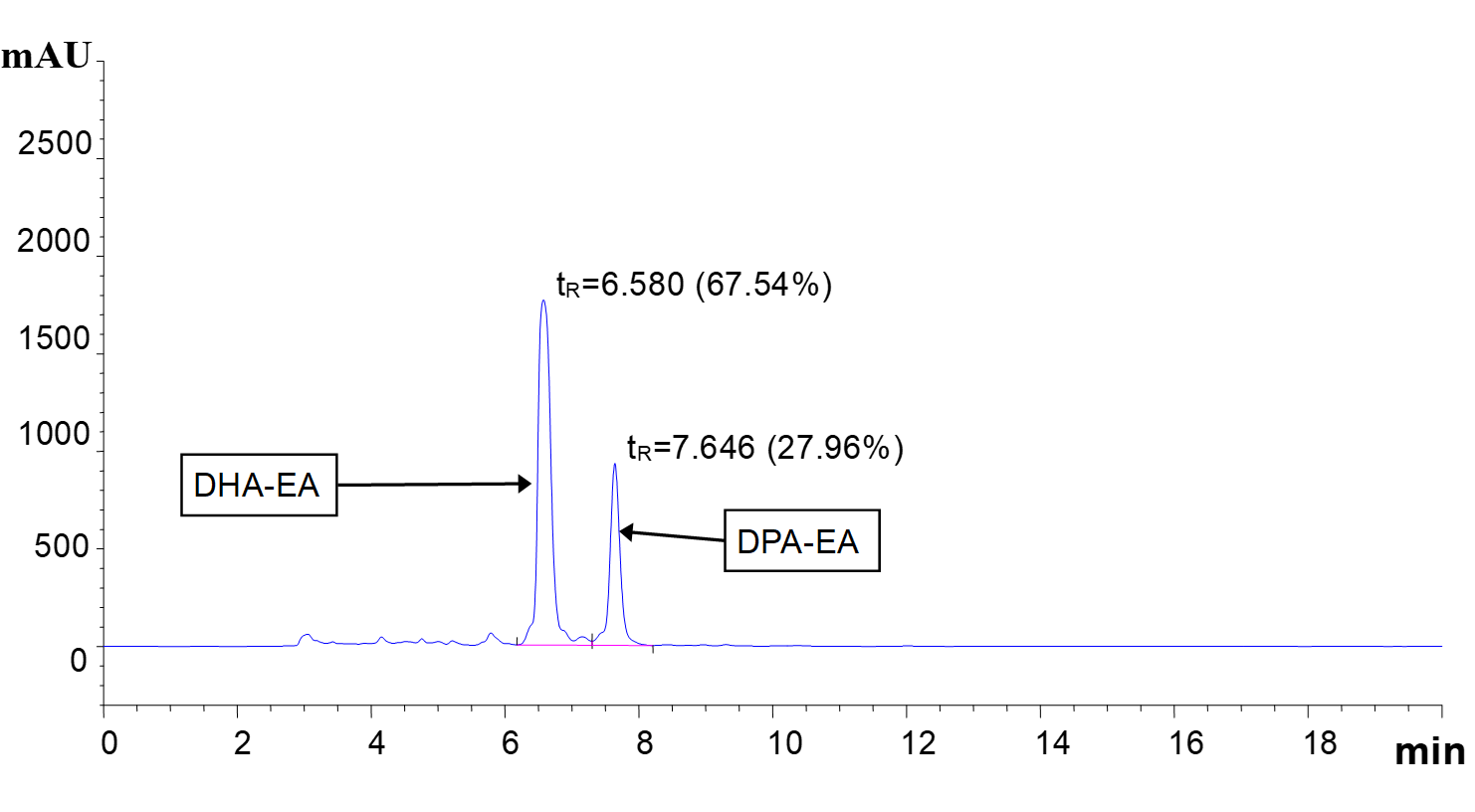
**

**Figure S1**. High-performance liquid chromatogram of PUFA-EA. The sample was analyzed on ODS2 column (250 mm × 4. 6 mm, 5 μm) with a mixture of methanol and H_2_O (90:10, *v/v*) as the mobile phase at a flow rate of 1 mL/min. The detection wavelength was 205 nm and the column temperature was 30 ^o^C.

**###**

*******

*******

*******

******

**Figure S2**. Quantification of Oil Red O staining (Fig. 1C) using Image J Pro Plus 6.0 in each group. Int den stands for integrated density.


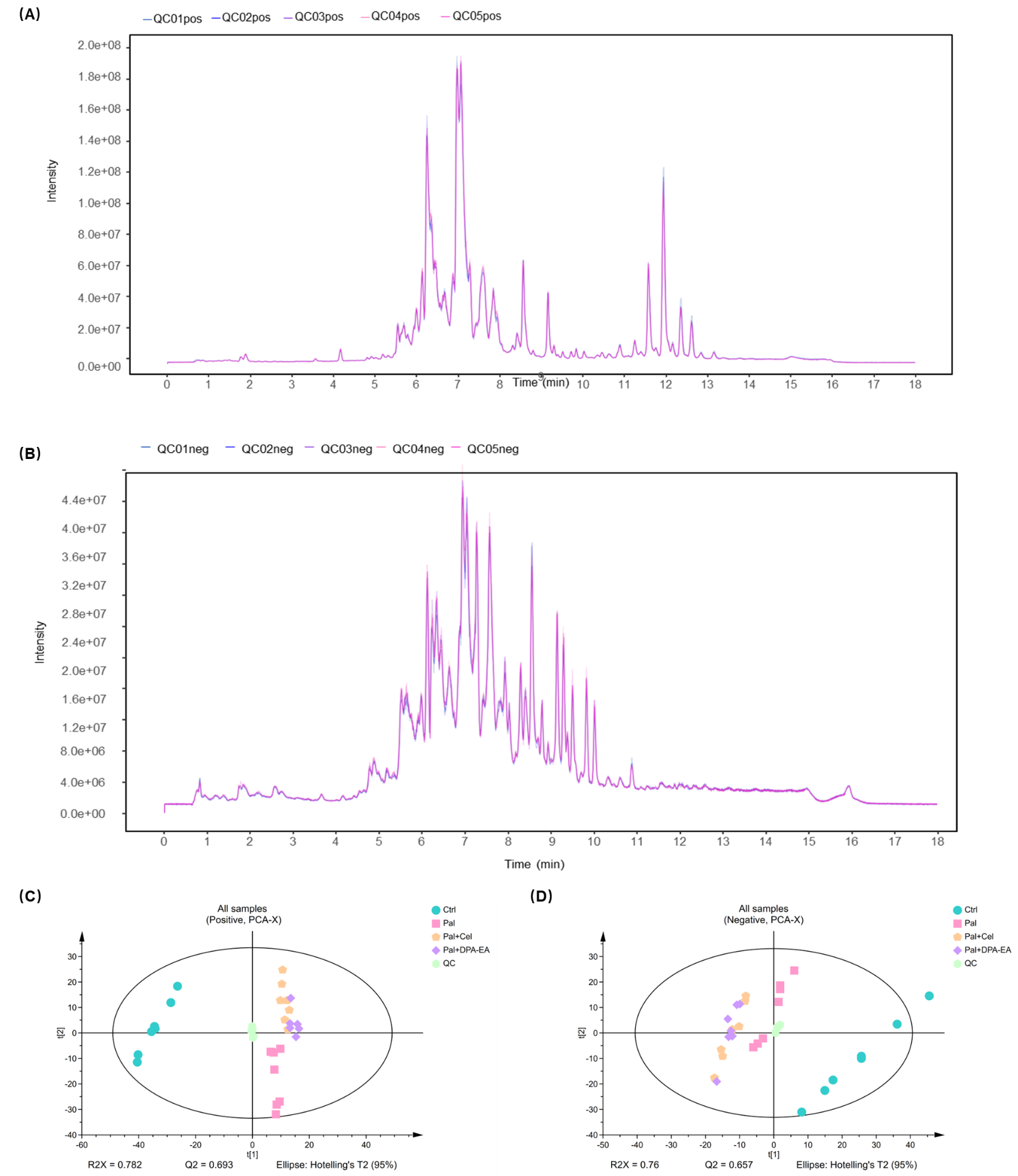


**Figure S3**. (A-B) Total ionization chromatography (TIC) of mixed samples. (A) is for TIC diagram in positive ion mode. (B) is for TIC diagram in negative ion mode. The ordinate indicates intensity (CPS), the abscissa indicates retention time(min). (C-D) PCA model analysis of all samples detected in Positive ion mode (C) and Negative ion mode (D).


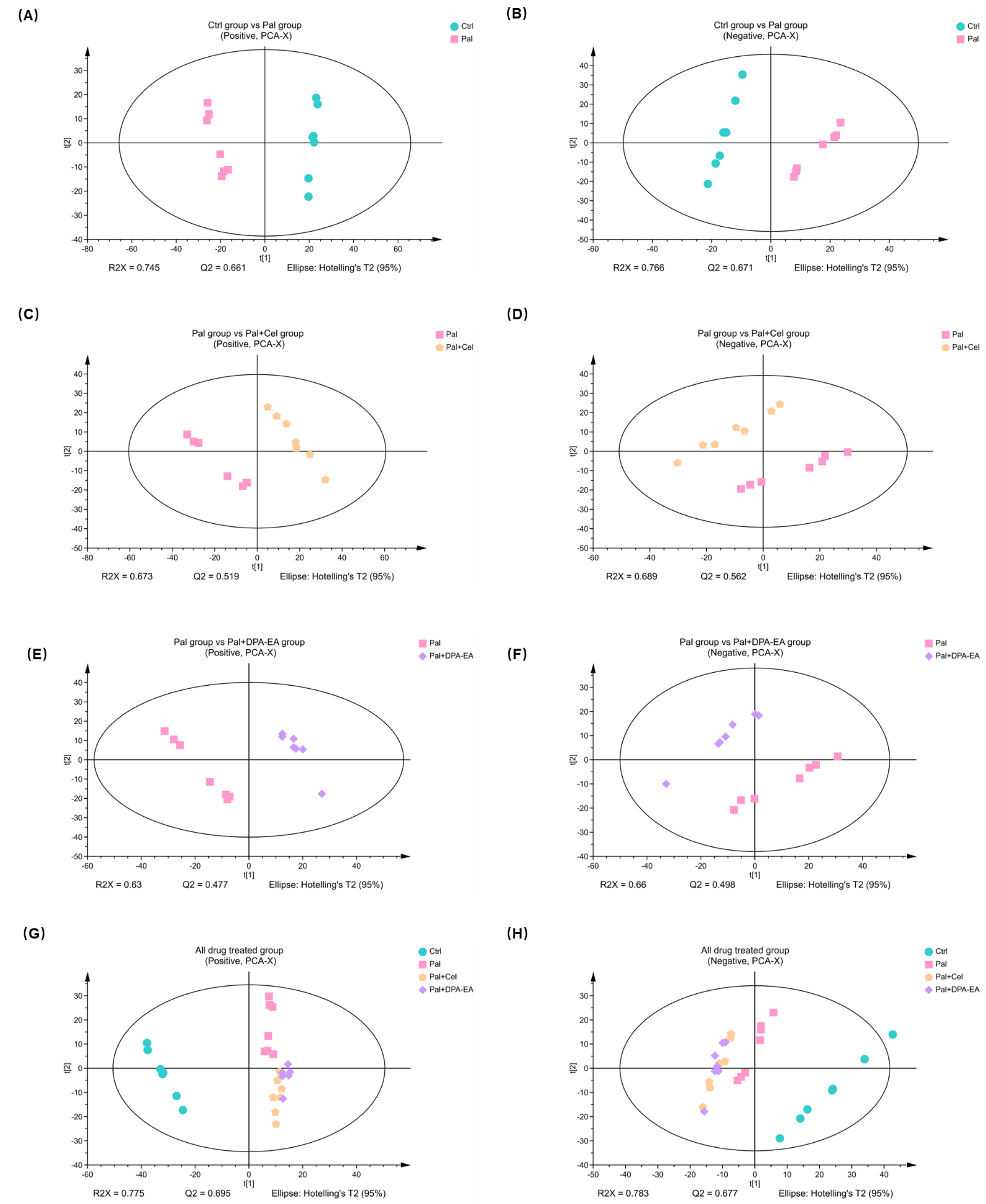


**Figure S4**. PCA model analysis of Pal group *versus* Ctrl group, Pal+Cel group *versus* Pal, Pal+DPA-EA group *versus* Pal group and all drug treated groups detected in Positive ion mode (A, C, E, G) and Negative ion mode (B, D, F, H), respectively.


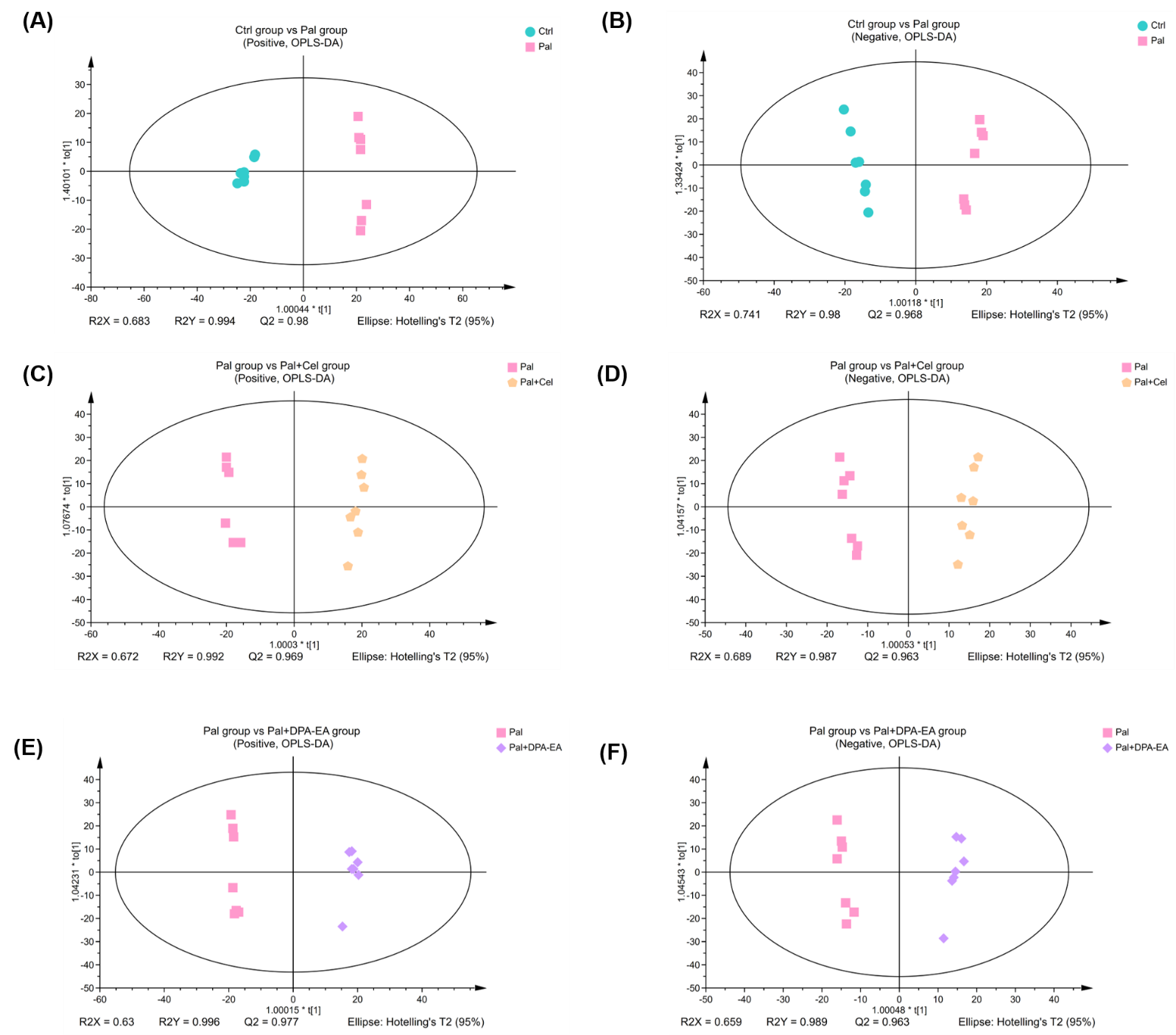


**Figure S5.** OPLS-DA score plots in each group for differential expressed lipid substances in positive (A, C and E) and negative (B, D and F) modes respectively. Ctrl groups *versus* Pal groups (A-B), Pal groups *versus* Pal+Cel groups (C-D) and Pal group *versus* Pal+DPA-EA group (E-F), respectively.

**
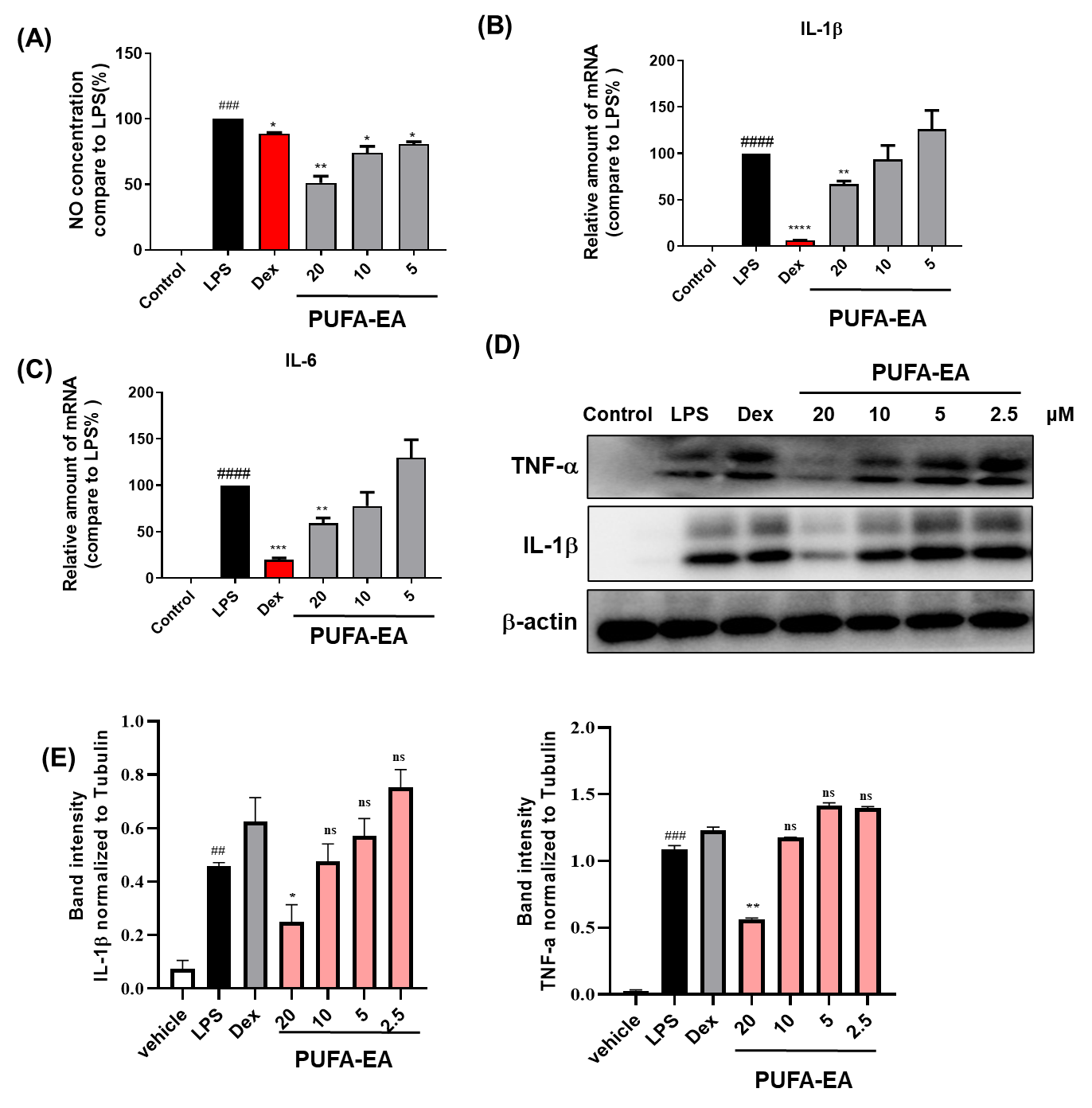
**

**Figure S6**. PUFA-EA dose-dependently inhibits IL-6, IL-1β, NO, and TNF-α productions in LPS-stimulated RAW264.7 cells. (A) The NO levels in the medium. (B) The mRNA expression levels of IL-1β in RAW264.7 cells. (C) The mRNA expression levels of IL-6 in RAW264.7 cells. (D) The protein expression levels of TNF-α and IL-1β in RAW264.7 cells. Macrophages were plated for 24 h and then challenged with LPS (1 μg/mL) with or without PUFA-EA at different concentrations. (E) grayscale analysis of IL-1β and TNF-α in Figure 9D of main text, respectively. NO levels in the medium were determined by the Griess assay. The mRNA expression levels of IL-1β, and IL-6 and the protein expression levels of TNF-α and IL-1β in RAW264.7 cells were determined by qPCR and Western Blot, respectively. Data are expressed as fold change relative to control values (samples treated with LPS alone), mean ± SEM (n ≥ 3). Student's t-test was used for the statistical analysis, #p < 0.05, ##p < 0.01, ###p < 0.001 compared with the control group; *p < 0.05 and **p < 0.01 (*vs*. LPS alone-stimulated group).


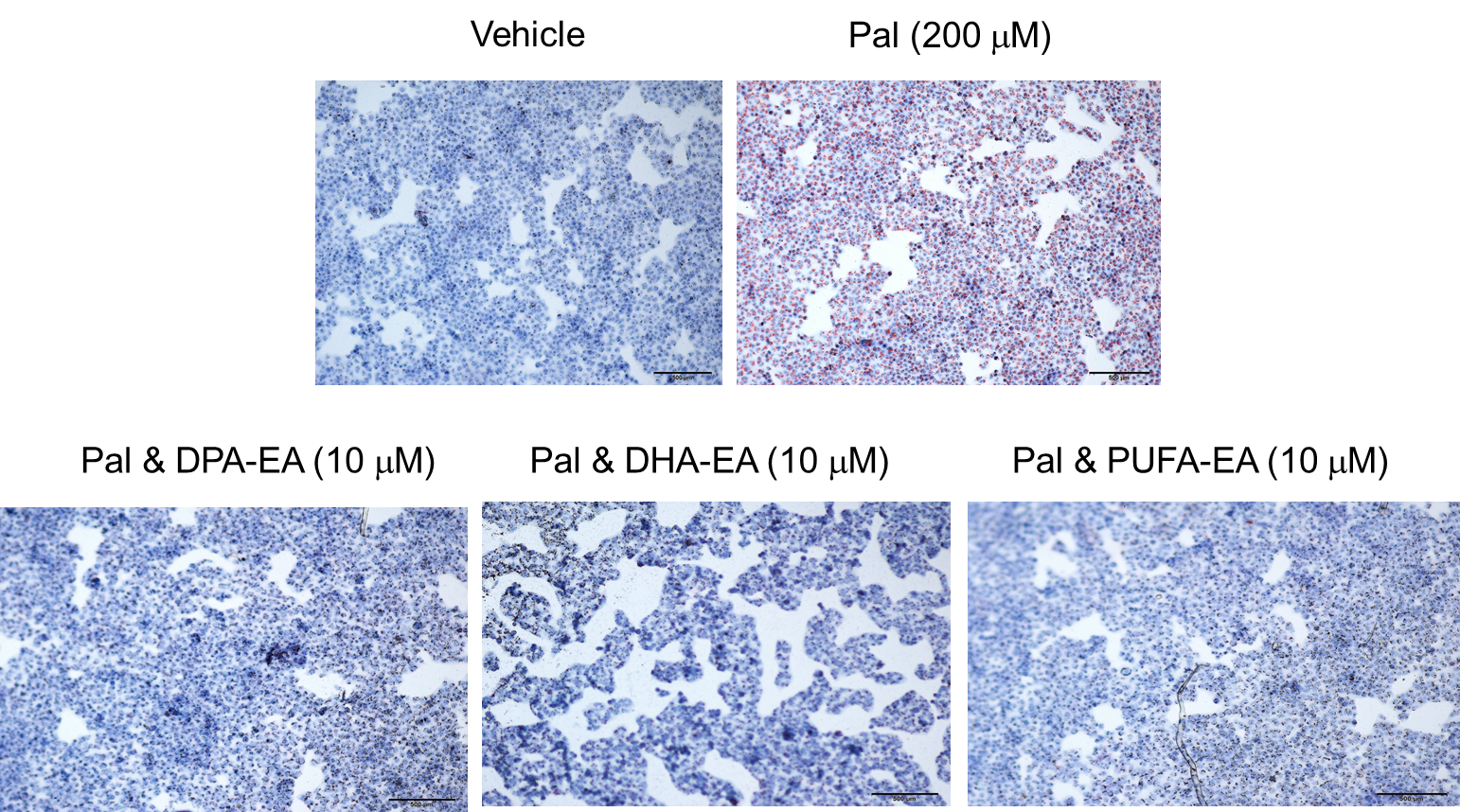


**Figure S7**. Assessment of the lipid-lowering effects of selected samples in Pal-stimulated HepG2 cells using Oil red staining assay. Images were captured with a microscope (100×).


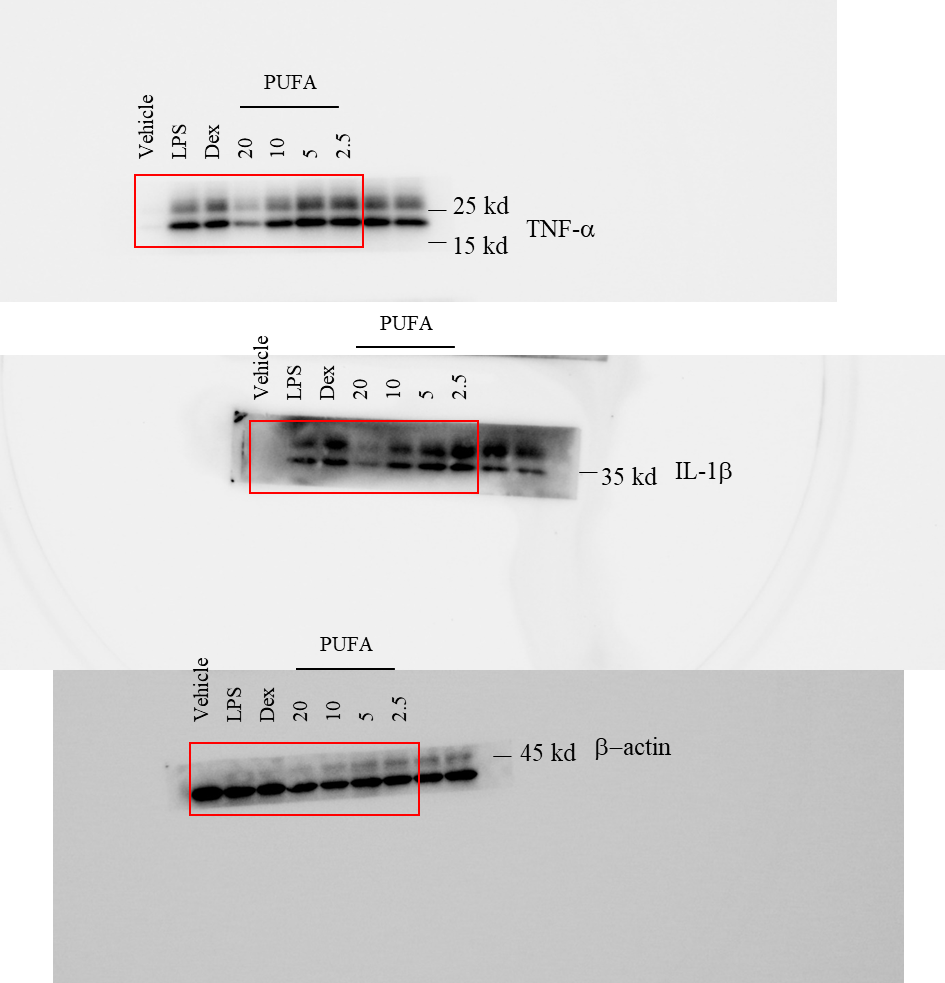


**Figure S8**. Original pictures of western blot in Figure 9D.
